# Fungal canker pathogens trigger carbon starvation by inhibiting carbon metabolism in poplar stems

**DOI:** 10.1038/s41598-019-46635-5

**Published:** 2019-07-12

**Authors:** Ping Li, Wenxin Liu, Yinan Zhang, Junchao Xing, Jinxin Li, Jinxia Feng, Xiaohua Su, Jiaping Zhao

**Affiliations:** 1 State Key Laboratory of Tree Genetics and Breeding, Forestry Institute of New Technology, Chinese Academy of Forestry, Beijing, China; 2 State Key Laboratory of Tree Genetics and Breeding, Institute of Forestry, Chinese Academy of Forestry, Beijing, China

**Keywords:** RNA sequencing, Plant physiology, Biotic

## Abstract

Carbon starvation is the current leading hypothesis of plant mortality mechanisms under drought stress; recently, it is also used to explain tree die-off in plant diseases. However, the molecular biology of the carbon starvation pathway is unclear. Here, using a punch inoculation system, we conducted transcriptome and physiological assays to investigate pathogen response in poplar stems at the early stages of *Botryosphaeria* and *Valsa* canker diseases. Transcriptome assays showed that the majority of differentially expressed genes (DEGs) in stem phloem and xylem, such as genes involved in carbon metabolism and transportation, aquaporin genes (in xylem) and genes related to the biosynthesis of secondary metabolites and the phenylpropanoid pathway (related to lignin synthesis), were downregulated at 7 days after inoculation (DAI). Results also showed that the expression of the majority of disease-resistance genes upregulated in poplar stems, which may be connected with the downregulation expression of the majority of WRKY family genes. Physiological assays showed that transpiration rate decreased but WUE (water use efficiency) increased the 3 and 7 DAI, while the net photosynthetic rate decreased at 11 DAI in *Botryosphaeria* infected poplars (ANOVA, P < 0.05). The NSC (non-structural carbohydrates) content assays showed that the soluble sugar content of stem phloem samples increased at 3, 7, and 11 DAI that might due to the impede of pathogen infection. However, soluble sugar content of stem xylem and root samples decreased at 11 DAI; in contrast, the starch content unchanged. Therefore, results revealed a chronological order of carbon related molecular and physiological performance: declination of genes involved in carbon and starch metabolism first (at least at 7 DAI), declination of assimilation and carbon reserve (at 11 DAI) second. Results implied a potential mechanism that affects the host carbon reserve, by directly inhibiting the expression of genes involved in carbon metabolism and transport.

## Introduction

*Populus* spp. are important model species in biotechnology, and one of the top three major afforestation tree species in China. *Botryosphaeria* and *Valsa* canker diseases of poplar are related to water deficit and exacerbated by high temperature and drought stress^1–5^. In the context of global climate change, these two canker diseases occur almost annually in poplar plantations in China. In 2010, over 0.85 million hectares of poplar plantations in China suffered *Botryosphaeria*, *Valsa*, and *Coryneum* canker diseases^6^. Researchers have focused on the epidemiology and management of, host response to, and interactions between the pathogens and environmental factors of poplar canker diseases^7,8^. However, the mechanism underlying tree mortality caused by canker pathogens is unclear.

Carbon starvation may explain tree mortality under drought and high-temperature stresses^9^. Under drought conditions, water-deficit signals induce stomatal closure to prevent hydraulic failure, and the resulting lower stomatal conductance reduces photosynthetic carbon uptake, decreased photosynthesis, leading to carbon depletion in sink tissue (including stems, root, flowers, etc.), and continued metabolic demand for carbohydrates^10^. In pathogen-associated mortality, drought stress may change the demographics of pathogens^9^; pathogens subsequently drive forest mortality independently or in conjunction with drought-induced changes in the host’s physiological condition^11,12^. Pathogen infections also significantly alter physiological performance, such as inhibiting stomatal opening and the photosynthetic rate, thus triggering physiological changes similar to those triggered by drought stress^13–15^. This, combined with the increased demand for energy and carbon for disease resistance, depletes the carbon reserve in the sink tissues^16^. Because the lesion or necrosis sites initially develop in bark, we suspected that the occur of poplar canker diseases could impede carbohydrate transport in phloem, and then affect the carbon reserve in stems and roots. Using a completely-girdling-inoculation system, we investigated host physiology and carbon metabolism at the middle stage of *Botryosphaeria* and *Valsa* canker diseases. Obstacles to carbohydrate transport and carbon depletion occurred in stem xylem, rather than being related to a hydraulic constraint, which is related to poplar mortality (Li, Xing *et al*. 2019; under review).

In addition to depleting carbon reserves by inhibiting photosynthesis, pathogens also induce carbon starvation by directly depleting carbon reserves, accelerating carbon consumption, and increasing repair costs^16^. Rust infections of poplar significantly alter photosynthesis and respiration, and photosynthesis and respiration are important components of the response of poplar to pathogen^17^. Transcriptome analysis reflected the demands of defense during rust infestation through changes in photosynthesis, respiration, and carbohydrate metabolism^17^. High-throughput sequencing methods, such as microarray assays^18^, analysis of metabolites by gas chromatography-mass spectrometry^19^, micro RNA (miRNA) sequencing^20^, and transcriptomics^21,22^ have been applied to the study of poplar canker diseases. However, aimed to control and management of diseases, genes related to disease resistance were mainly discussed in above researches. Therefore, the molecular biology of both the photosynthetic and respiratory responses of poplar to infection with canker pathogens and their effects on host carbon metabolism and transportation must be investigated.

To assess the effect of canker pathogens on carbon metabolism and transportation in poplar, we used a punch inoculation system, coupled with transcriptomic, gas exchange, and non-structural carbohydrates (NSCs, mainly including the soluble sugars and starch) content analyses at the early stage of *Botryosphaeria* and *Valsa* canker diseases of poplar. Xylem is the battleground for plant hosts and vascular wilt pathogens^23^. As describe above, the stem xylem tissue is also related to disease resistance, carbon metabolism, and water transport in canker diseases. Therefore, stem phloem and xylem tissues from around inoculation sites (which are also carbohydrate sink tissues) were used in this study.

## Results

### Global review of transcriptome sequencing data

RNA sequencing (RNA-seq) generated an average of 84,286,700 clean reads, from a minimum of 70,023,048 (Control xylem sample 2, Ctrl_Xy_2) to a maximum of 89,642,948 (*Botryosphaeria dothidea* inoculated phloem sample 1, Bdo_Ph_1). The clean reads comprised at least 9.7 GB. The number of clean bases per sample was at least 19.87-fold the number in the poplar genome (~500 MB)^24^, which is sufficient for gene expression analysis. More than 52% of the clean reads in each library, (35,589,872 [*Valsa sordida* inoculated xylem sample 2, Vso_Xy_2] to 49,835,656 [*V*. *sordida* inoculated phloem sample 2, Vso_Ph_2] reads) were mapped to the reference *P*. *trichocarpa* genome (Table 1).

**Table 1:** Illumina RNA-Seq output statistics of poplar stems infected with canker pathogens.

ID	Raw Reads Number	Clean Reads Number	Clean Reads Rate (%)	Clean Bases Number	Total Reads	Mapped Reads	Mapping Rate	MultiMap Reads	MultiMap Rate (%)
Vso_Ph_1	48,423,943	47,416,356	97.92	11,932,758,024	85,225,692	49,835,656	58%	2,692,108	0.03
Vso_Ph_2	50,367,634	49,364,704	98.01	12,423,211,270	88,107,330	52,281,364	59%	2,811,085	0.03
Bdo_Ph_1	58,760,201	58,626,888	99.77	14,759,035,740	89,272,184	48,525,403	54%	2,697,157	0.03
Bdo_Ph_2	51,400,883	49,046,657	95.42	12,342,161,406	82,748,230	44,622,382	54%	2,546,407	0.03
Ctrl_Ph_1	79,530,164	79,275,154	99.68	19,959,391,628	87,512,254	51,855,440	59%	3,004,736	0.03
Ctrl_Ph_2	67,574,052	67,415,778	99.77	16,965,704,828	85,082,502	48,299,231	57%	3,006,199	0.04
Vso_Xy_1	66,189,963	66,030,811	99.76	16,617,621,932	82,442,750	35,589,872	43%	1,732,008	0.02
Vso_Xy_2	44,267,940	43,537,925	98.35	10,958,415,320	78,253,422	35,412,647	45%	1,873,055	0.02
Bdo_Xy_1	61,763,767	61,545,144	99.65	15,487,160,550	89,642,948	40,213,639	45%	2,208,309	0.02
Bdo_Xy_2	62,464,972	62,210,959	99.59	15,646,188,196	83,910,706	37,663,575	45%	1,830,723	0.02
Ctrl_Xy_1	52,998,345	52,869,016	99.76	13,307,448,344	89,219,344	42,150,946	47%	2,296,483	0.03
Ctrl_Xy_2	39,265,510	38,610,488	98.33	9,715,310,208	70,023,048	37,230,574	53%	1,731,286	0.02
Average	56,917,281	56,329,157	98.83	14,176,200,621	84,286,700	43,640,061	52%	2,369,130	0.03

**Figure 1:**
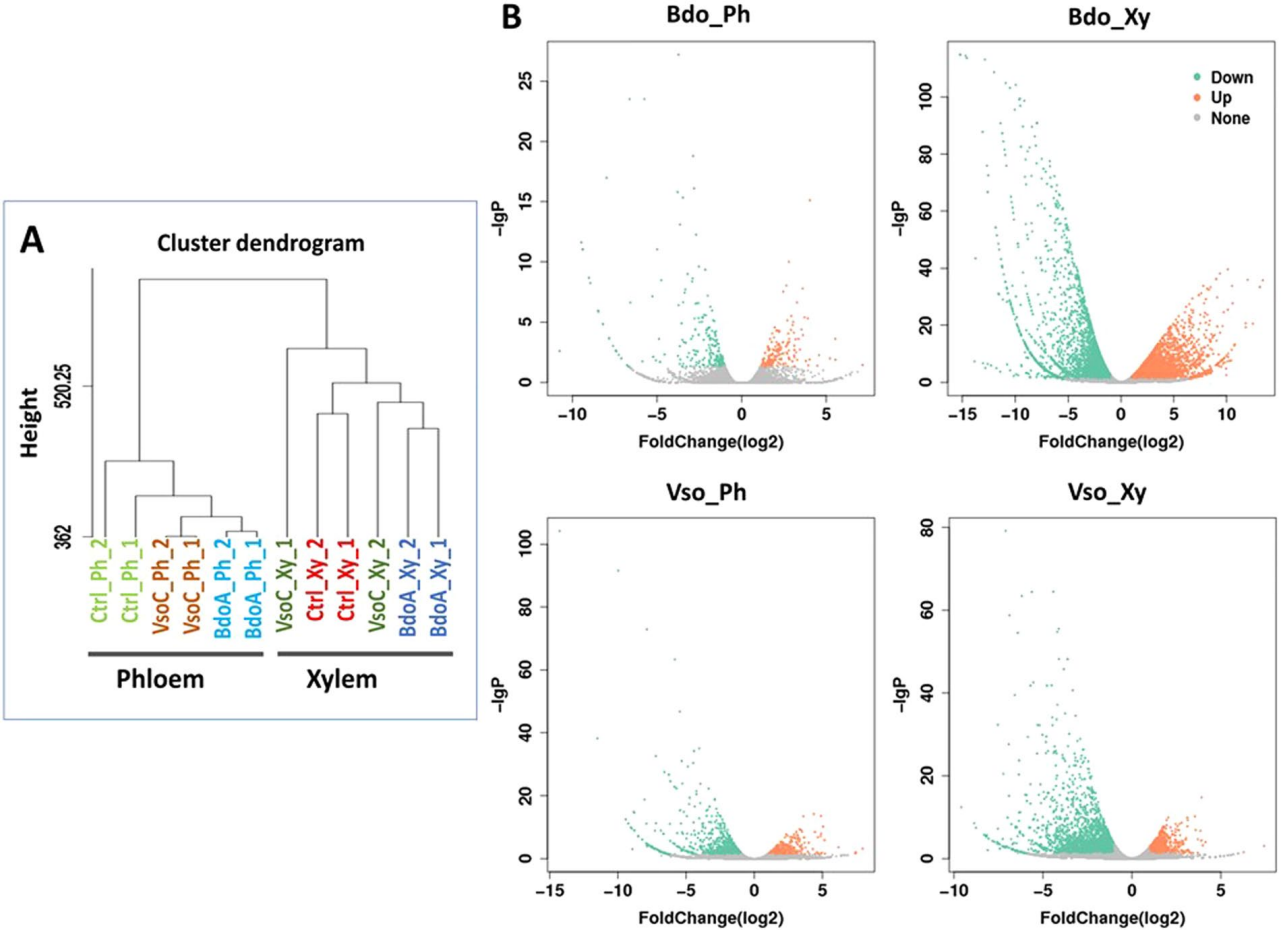
Global evaluation of transcriptome sequencing data of poplars infected by *Botryosphaeria* and *Valsa* canker pathogen. (**A**) Hierarchical clustering analysis of gene expression shown the consistency of two biological replicates in 6 groups (Bdo_Ph, Bdo_Xy, Vso_Ph, Vso_Xy, Ctrl_Ph and Ctrl_Xy). (**B**) Volcano map showing that the majority of DEGs are downregulated in pathogen-infected poplar stems. DEGs were selected using log_2_FC≥ or ≤−1 and FDR ≤ 0.05 including those only expressed in one library, and FDR < 0.05. Bdo_Ph, Bdo_Xy, Vso_Ph and Vso_Xy represent these four treatments compared to their controls, respectively.

RPKM expression data of 12 poplar samples were listed in Supplementary Datasets 1. Correlation analysis of whole-genome gene expression was used to evaluate the consistency of the sequencing data. The Pearson coefficients of two biological replicates were >0.88 with the exception of the Vso_Xy group. The consistency value of samples Vso_Xy_1 and Vso_Xy_2 was 0.70, lower than that (0.8) recommended for RNA-seq analysis (Supplementary Table S1). A hierarchical clustering analysis was performed to evaluate the consistency of the RNA-seq data. In the phloem group, two Bdo_Ph samples and two Vso_Ph samples clustered together, respectively; these two subgroups were equidistant from the phloem control samples (Ctrl_Ph). In xylem, two Bdo_Xy (*B*. *dothidea* inoculated xylem sample) and two Ctrl_Xy samples clustered together; however, one *Valsa*-inoculated sample (Ctrl_Xy_1) was distant from the other samples (Fig. 1A). Therefore, only Ctrl_Xy_2 was representative of the gene expression pattern of *Valsa*-infected poplar. Ctrl_Xy_1 was thus omitted from subsequent analyses.

### Gene expression patterns in poplars infected with canker pathogens

Totals of 375, 2,945, 1,118, and 1,535 differentially expressed genes (DEGs) were detected between Bdo_Ph, Bdo_Xy, Vso_Ph, and Vso_Xy, respectively, and the untreated controls (Table 2 and Supplementary Datasets 2). Interestingly, the number of downregulated genes was significantly greater than that of upregulated genes (chi-squared test, *P* < 0.05), with the exception of Bdo_Ph *vs*. Ctrl_Ph (chi-squared test, 0.05 < *P* < 0.10) (Fig. 1B). For example, 2,209 genes were downregulated in Bdo_Xy, three-fold the number upregulated (Table 2 and Supplementary Datasets 2). Transcription factors (TFs) regulate the expression of genes. The majority of TF-encoding genes were downregulated in xylem and phloem tissues (Supplementary Table S2). Totals of 24, 10, 25, and 18 TF-encoding DEGs (60.0%, 90.9%, 62.5%, and 69.2% of TF DEGs) in Bdo_Xy, Bdo_Ph, Vso_Xy, and Vso_Ph were downregulated, respectively. Therefore, the downregulation of the majority of DEGs might be due to downregulation of TF genes.

**Table 2:** Number of canker pathogens induced differentially expressed genes (DEGs) in the phloem and xylem of poplar stems.

Treatments	Bdo_Ph vs Ctrl_Ph	Bdo_Xy vs Ctrl_Xy	Vso_Ph vs Ctrl_Ph	Vso_Xy vs Ctrl_Xy
Number of up-regulated genes (Nu)	164	736	389	598
Number of down-regulated genes (Nd)	211	2,209	729	937
Ratio of Nd/Nu	1.29	3.00**	2.22**	1.57**
Total number of differentially expressed genes	375	2,945	1,118	1,535
Percentage of down-regulated genes in total (%)	56.3	72.5	65.2	61.0

Notation: The DEGs were selected using log2(FC)≥ or ≤−1 and FDR ≤ 0.05 including those only expressed in one library, and FDR < 0.05. Asterisks indicate the number of down-regulated genes (Nd) is more than that of up-regulated genes (Nu) (Chi-square test, null hypothesis is Nd = Nu; *P < 0.05; **P < 0.01).

**Figure 2:**
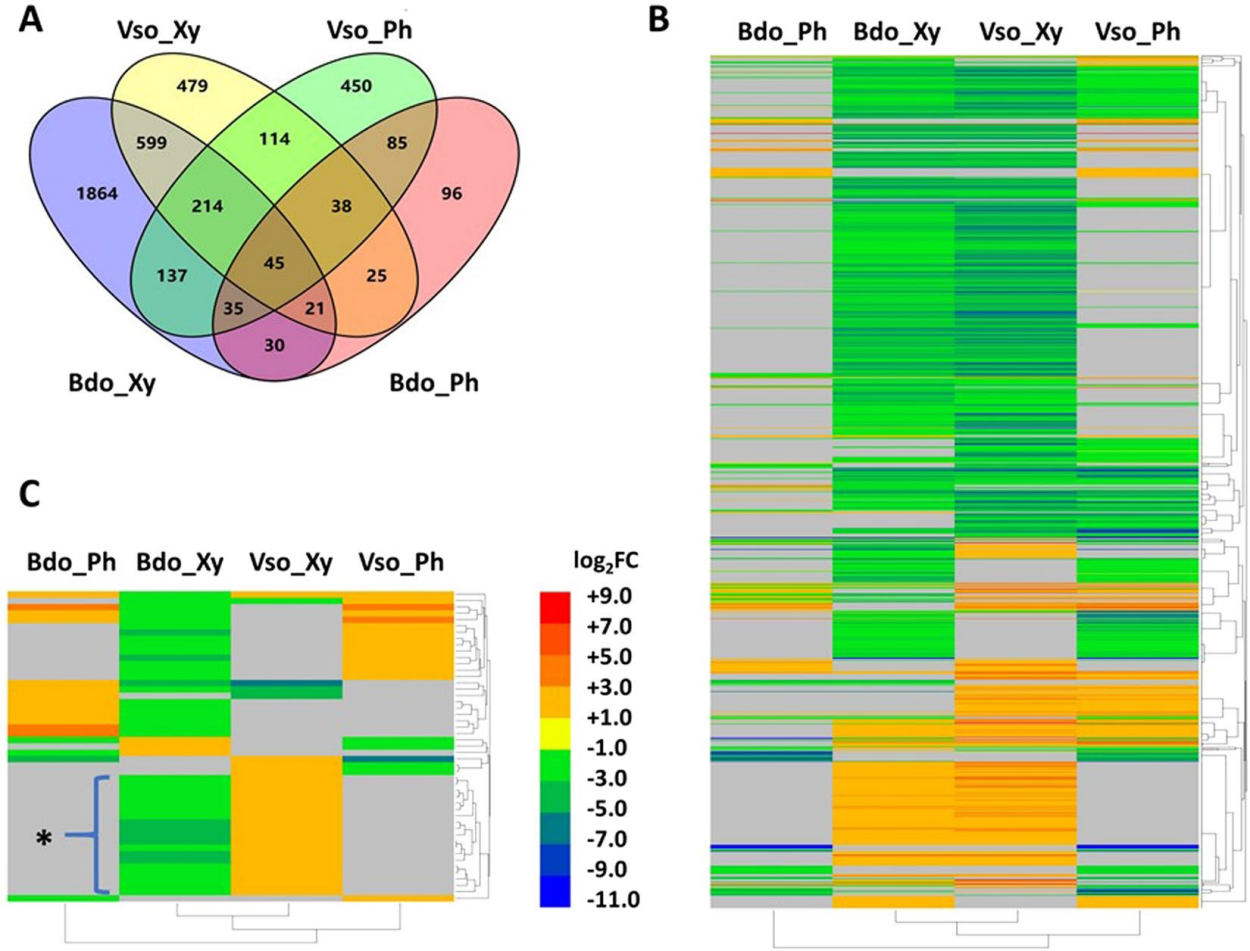
Co-expressed DEGs in stems of poplar infected with pathogen *Botryosphaeria* and *Valsa*. (**A**) Venn diagram of DEGs in phloem and xylem of *Botryosphaeria* and *Valsa* infected poplar stems. (**B**) Heatmap of co-expressed DEGs in phloem and xylem of *Botryosphaeria* and *Valsa* infected poplar stems. (**C**) Heatmap of divergently regulated poplar DEGs. For the DEG that only detected in pathogen treatments library or in control library, their expression (log_2_FC value) was set as 8.0 and −11.0 respectively because the log_2_FC value of the most up-regulated DEG (Potri.013G126500, function unknown) was 7.95 and the log_2_FC value of the most down-regulated gene (Potri.008G101600, encoding gibberellin 2-beta-dioxygenase) was −10.97. Grey indicates genes did not significantly differentially changed in this treatment. Asterisk in panel C indicates 19 divergently regulated DEGs that only be detected in xylem of pathogen *Botryosphaeria* and *Valsa* infected poplars.

A total of 4,332 DEGs were detected in the xylem and phloem of *Botryosphaeria*- and *Valsa*-infected poplars. Among them, 2,889 DEGs were detected in only one of the four comparisons, and 1,343 DEGs in at least two comparisons (Fig. 2A and Supplementary Datasets 3). The direction of the change in expression of most of the coexpressed DEGs (1,294 of 1,343 genes) was the same in at least two comparisons (Fig. 2B); however, this was not the case for 49 of the DEGs (Supplementary Datasets 3). Nineteen DEGs were coexpressed only in xylem tissue (Bdo_Xy and Vso_Xy) and were downregulated by *Botryosphaeria* but upregulated by *Valsa* (Fig. 2C); these genes encoded proteins such as laccase, peroxidase, lysine-rich arabinogalactan protein, fasciclin-like arabinogalactan protein, E3 ubiquitin-protein ligase, and LRR receptor-like serine/threonine protein kinase. Therefore, poplar responds to the two canker pathogens in different ways.

### Functional analysis of DEGs

A KEGG enrichment analysis^25^ showed that the DEGs in Bdo_Xy, Vso_Ph, and Vso_Xy were significantly enriched in at least three metabolic pathways, but the DEGs in Bdo_Ph were not enriched in any pathway (possibly because of the small number of DEGs in this category [n = 375]). In Bdo_Xy, for example, 133, 40, and 29 DEGs were enriched in the biosynthesis of secondary metabolites, starch and sucrose metabolism, and phenylpropanoid biosynthesis pathways, respectively (Supplementary Table S3). To reveal the molecular mechanisms of carbon starvation that induced by canker pathogens, the expression patterns of genes related to carbohydrate metabolism and water transportation were evaluated, moreover, the expression patterns of genes related to disease resistance or pathogenesis were also assessed.

### Genes involved in starch and sucrose metabolism

**Figure 3:**
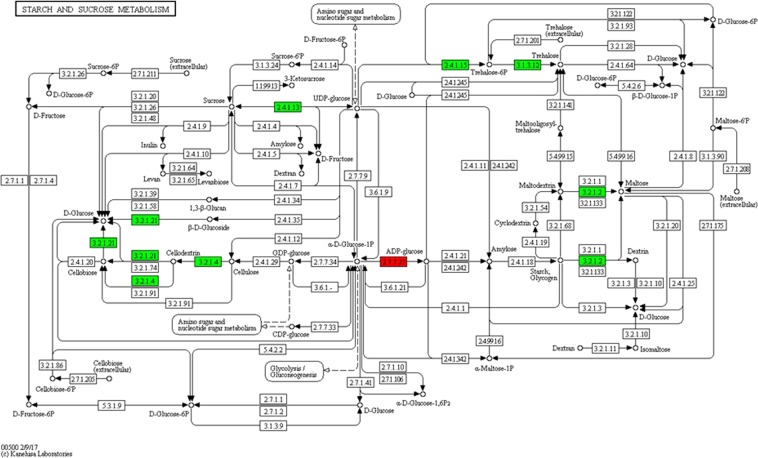
DEGs related to starch and sucrose metabolism were downregulated by pathogen *Botryosphaeria*. KEGG pathway map for starch and sucrose pathway (ko00500). Red indicates activity of the enzyme is induced by canker pathogen, while green indicates activity of the enzyme is inhibited.

KEGG enrichment analysis revealed that 40 DEGs in Bdo_Xy were involved in starch and sucrose metabolism (Supplementary Table S3). The expression of 10 DEGs related to the biosynthesis of D-glucose, cellobiose, cellodextrin, sucrose, trehalose, trehalose-6P, maltose, and dextrin was downregulated, but that of a glucose-1-phosphate adenylyltransferase-encoding gene (involved in production of ADP-glucose) was upregulated (Fig. 3). Based on the results of functional annotation in NCBI, SWISS-PROT and GO ontology database, more genes (73) related to starch and sucrose metabolism and associated with transport of sugars were detected in Bdo_Xy; 56 of them were downregulated (Table 3). DEGs encoding sucrose synthase, amylase, trehalose-phosphate synthase, and pectinesterase were downregulated, which would result in decreased D-glucose, cellobiose, sucrose, trehalose-6P, dextrin, and maltose contents in xylem tissue and increased ADP-glucose content. Therefore, these results implied that *Botryosphaeria* infection could decrease the levels of sucrose, starch, and other carbohydrates in poplar stems.

**Table 3:** DEGs related to starch and sucrose metabolism in poplar xylem tissues infected with canker pathogens.

	Bdo_Xy vs Ctrl_Xy		Vso_Xy_Ctrl_Xy	
	Gene Name	Log_2_FC	Gene Name	Log_2_FC
Galacturonosyltransferase	Potri.014G073800	−1.10	Potri.016G001700	1.45
	Potri.001G416800	−2.21	Potri.007G031700	2.14
	Potri.011G132600	−3.30	Potri.010G129400	2.76
	Potri.017G106800	−1.22	Potri.002G200200	2.24
	Potri.014G040300	−1.05	Potri.008G192600	3.46
	Potri.014G125000	−1.55		
Beta-amylase	Potri.008G174100	−2.00	—	—
Alpha-xylosidase and Beta-D-xylosidase	Potri.010G141400	−3.27	Potri.010G141400	−5.03
	Potri.002G197200	−1.24	Potri.008G120000	−2.75
Beta-glucosidase and Glucan endo-1,3-beta-D-glucosidase	Potri.008G094200	−2.55	Potri.008G094200	−2.58
	Potri.011G094400	−3.12	Potri.011G094400	−2.39
	Potri.010G142800	−3.75	Potri.010G142800	−3.96
	Potri.001G206800	−2.50	Potri.001G255100	−9.33
	Potri.001G222900	−2.41	Potri.T167100	−5.85
	Potri.004G109200	Down	Potri.016G057400	−3.57
	Potri.015G041300	−2.14	Potri.016G057600	4.49
	Potri.004G019300	1.61		
	Potri.004G019700	1.9		
	Potri.004G019400	1.96		
	Potri.004G019800	1.24		
	Potri.008G056000	−1.84		
	Potri.001G380600	−2.1		
	Potri.008G055900	−1.85		
	Potri.014G158400	−1.06		
	Potri.017G130200	1.02		
	Potri.014G184600	Down		
Endoglucanase	Potri.003G139600	−2.52	Potri.003G139600	−3.39
	Potri.008G079500	−3.71	Potri.008G079500	−6.45
	Potri.005G237700	−2.3	Potri.019G069300	−2.16
	Potri.014G157600	−2.43	Potri.003G147600	−4.10
	Potri.009G123900	−2.19	Potri.001G083200	−4.40
	Potri.001G092200	−1.79		
Glucose-1-phosphate adenylyltransferase	Potri.005G229700	1.06		
	Potri.014G171800	1.01		
Pectinesterase	Potri.001G162400	−1.82	Potri.001G162400	−1.86
	Potri.015G127700	−5.36	Potri.003G072700	2.10
	Potri.011G135000	−1.93		
	Potri.002G202600	−2.23		
	Potri.014G127000	−2.41		
	Potri.006G134500	−1.60		
Sucrose synthase	Potri.018G063500	−2.24		
	Potri.006G136700	−1.62		
Trehalose-6-phosphate and trehalose-phosphate synthase	Potri.015G126900	−3.80	Potri.015G126900	−4.19
	Potri.003G112400	−4.37	Potri.003G112400	−5.51
	Potri.005G077200	−2.47	Potri.005G077200	−3.23
	Potri.003G094500	1.04	Potri.012G126100	2.82
	Potri.001G139500	1.06		
	Potri.007G090900	−1.53		
UDP-glucose 6-dehydrogenase	Potri.004G118600	−2.47		
	Potri.017G092000	−1.32		
	Potri.008G094300	−2.87		
UDP-glucuronate 4-epimerase	Potri.006G178500	−2.36	Potri.018G100400	1.52
	Potri.017G059100	−1.82		
Fructose-bisphosphate aldolase	Potri.006G165700	−1.15	Potri.006G165700	−1.44
	Potri.018G090100	−1.47		
	Potri.004G162400	1.33		
	Potri.007G015500	1.70		
Glucose-1-phosphate adenylyltransferase	Potri.005G229700	1.06		
	Potri.014G171800	1.02		
Glucose-6-phosphate 1-dehydrogenase and Glucose-6-phosphate/phosphate translocator	Potri.004G019900	2.46	Potri.004G019900	3.30
	Potri.005G006100	1.42		
	Potri.001G337400	−1.11		
Bidirectional sugar transporter SWEET	Potri.005G187300	−1.78		
	Potri.013G013800	−3.11		
	Potri.013G014400	−1.99		
	Potri.008G220600	2.26		
	Potri.001G355500	1.05		
Sugar transport protein	Potri.002G095900	−1.48	Potri.002G095900	−2.77
	Potri.010G089800	−4.27	Potri.002G212900	−2.60
	Potri.005G039900	−2.37		
UDP-galactose/UDP-glucose transporter	Potri.016G139100	−1.08		

Notation: The gene ID with the underline represents the gene co-expressed in Bdo_Xy and Vso_Xy.

**Figure 4:**
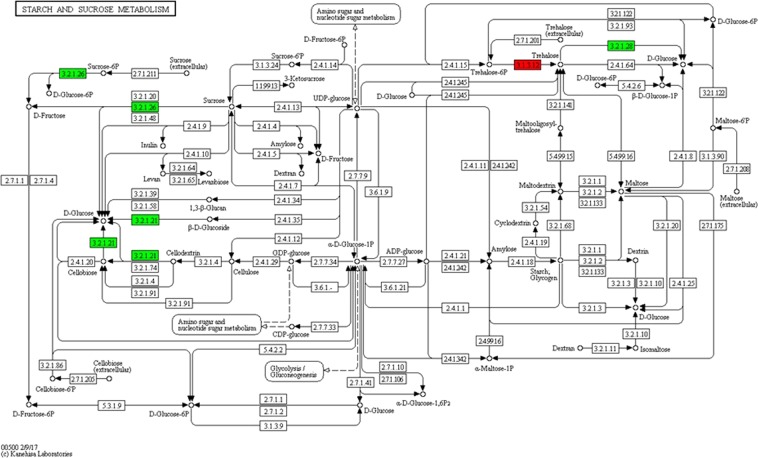
KEGG enrichment analysis of starch and sucrose pathway in stem xylem tissue of *Valsa*-infected poplar. KEGG pathway map for starch and sucrose pathway (ko00500). Red indicates activity of the enzyme is induced by canker pathogen, while green indicates activity of the enzyme is inhibited.

Infection with *Valsa* downregulated the expression of genes related to starch and sucrose metabolism. The genes encoding enzymes such as those that metabolize D-glucose, D-sucrose-6P, and D-fructose were downregulated, while that encoding trehalose was upregulated (Fig. 4). Functional analysis confirmed this result; 20 DEGs were downregulated and 10 upregulated in Vso_Xy. Notably, all five galacturonosyltransferase-encoding DEGs were upregulated by *Valsa* but downregulated by *Botryosphaeria* (Table 3).

### Genes involved in biosynthesis of secondary metabolites and the phenylpropanoid pathway

*Botryosphaeria* and *Valsa* significantly altered the expression of genes related to the biosynthesis of secondary metabolites. The 203 DEGs (encoding 85 categories of proteins) related to this pathway are listed in Supplementary Table S4. Of these 203 DEGs, 163 were downregulated and 41 upregulated in Bdo_Xy. Thus, canker pathogens suppressed the expression of the majority of genes related to secondary metabolism. For example, the expression of genes encoding proteins related to the biosynthesis of phenylpropanoids, flavonoids, and lignin was downregulated in Bdo_Xy (Supplementary Table S4).

Eighteen DEGs related to phenylpropanoid pathway (comprising one encoding 4-coumarate-CoA ligase, one encoding cinnamyl-alcohol dehydrogenase, and 16 [of 20] encoding peroxidases) were downregulated in Bdo_Xy. Additionally, 21 DEGs related to the phenylpropanoid pathway (comprising one encoding cinnamyl-alcohol dehydrogenase [also coexpressed in Bdo_Xy], six [of seven] encoding cinnamoyl-CoA reductases, and 14 [of 20] encoding peroxidases) were downregulated in Vso_Xy. Seven cinnamoyl-CoA reductase-encoding genes were expressed only in Vso_Xy and 15 peroxidase-encoding DEGs were coexpressed (identical direction of change) in Bdo_Xy and Vso_Xy (Supplementary Table S4). Plants produce several lignins (p-hydroxy-phenyl lignin, guaiacyl lignin, 5-hydroxy-guaiacly lignin, syringyl lignin) via the phenylpropanoid pathway; therefore, results implied that canker pathogens suppress the synthesis of lignins in poplar stems.

Cytochrome P450s play critical roles in the synthesis of lignins, various defense compounds, pigments, UV protectants, fatty acids, hormones, and signaling molecules. Several Cytochrome P450-encoding DEGs were detected in Bdo_Xy and Vso_Xy. The majority of these DEGs (20 of 32) were downregulated by *Botryosphaeria*, 14 of 18 were downregulated by *Valsa*, and 9 were co-expressed in Bdo_Xy and Vso_Xy (Supplementary Table S4). Moreover, 20 peroxidase-encoding DEGs were detected in Bdo_Xy and Vso_Xy respectively. The majority of these DEGs (16 of 20) were downregulated by *Botryosphaeria*, 14 of 20 were downregulated by *Valsa*, and 15 were co-expressed in Bdo_Xy and Vso_Xy. Therefore, fungal pathogens inhibit metabolism in xylem tissue at early stages of the pathogenesis of canker diseases of poplar.

### Disease resistance-related and aquaporin-encoding genes

Genes annotated as being related to disease resistance were upregulated by the two canker pathogens. For example, 8 of 12, 15 of 16, and 23 of 32 disease-resistance genes were upregulated in Bdo_Ph, Vso_Xy, and Vso_Ph, respectively, and 7 of 14 genes were upregulated in Bdo_Xy (Supplementary Table S5). WRKYs play important roles in plant immunity through SA, JA or ET pathway responses to various biotic stresses. Several WRKY transcription factors are involved in the response of poplar to canker pathogens. Results showed that the expression of the majority of the WRKY genes was downregulated in poplar, particularly in the two xylem tissues (Supplementary Table S6). For example, 20 of 23 and 21 of 28 WRKY genes were significantly downregulated in Bdo_Xy and Vso_Xy, respectively; compared to 2 of 7 and 5 of 11 WRKY genes in Bdo_Ph and Vso_Ph. As reviewed recently^26^, some “negative” regulatory WRKY genes (such as those of subfamilies WRKY11, WRKY40, WRKY7, and WRKY48) were downregulated by pathogens, whereas some WRKY genes (such as those of subfamilies WRKY22, WRKY53, WRKY33, and WRKY70) which had with “positive” regulatory role on plant resistance or disease response upregulated (Supplementary Table S6). In particular, 3 WRKY33 genes (Potri.016G128300, Potri.006G105300 and Potri.013G153400) upregulated in more than two treatments. Thus, the down- and upregulation of the above WRKY genes were consistent with the increased expression of the majority of disease resistance genes in host poplars. In addition, 3 WRKY75 genes (Potri.015G099200, Potri.012G101000 and Potri.003G169100), 1 WRKY28 gene (Potri.001G352400) and 2 WRKY31 genes (Potri.014G155100 and Potri.011G007800) downregulated in more than two treatments.

Fourteen aquaporin-encoding DEGs were detected in xylem and phloem during canker-pathogen infection (Table 4). Seven of eight aquaporin-encoding genes (one *PIP1*; *4*, *PIP2*; *3*, *PIP2*; *4*, *SIP1*; *2*, two *TIP1*; *1*, and two *TIP1*; *3* genes) were downregulated in Bdo_Xy, whereas five of seven genes (one *PIP2*; *3*, *TIP1*; *3*, and *TIP1*; *3*, and two *PIP2*; *2* genes) were downregulated in Bdo_Ph. Potri.016G089500, which encodes *PIP2*; *3*, was downregulated in both phloem and xylem by *Botryosphaeria* and *Valsa*. Potri.003G180900 (encoding *NIP6*; *1*), Potri.005G109200 (encoding *PIP2*; *7*), and Potri.009G127900 (encoding *PIP2*; *1*) were upregulated in one or both tissues (Table 4). Aquaporins are crucial for intercellular transport of water, and PIPs repair xylem embolisms under drought stress. Therefore, the downregulation of several aquaporin-encoding genes suggests that the canker pathogens inhibit recovery from xylem embolism.

**Table 4:** The differentially expressed Aquaporin-encoding genes in the phloem and xylem in canker pathogens inoculated poplar stems.

Gene	Subgroup	Bdo_Ph vs Ctrl_Ph	Bdo_Xy vs Ctrl_Xy	Vso_Ph vs Ctrl_Ph	Vso_Xy vs Ctrl_Xy
Potri.003G180900	NIP6;1	2.33	—	2.58	—
Potri.005G109200	PIP2;7	4.00	—	—	—
Potri.009G127900	PIP1;2	—	1.52	—	—
Potri.016G089500	PIP2;3	−1.54	−2.63	—	—
Potri.008G050700	TIP1;3	−2.81	−4.41	−4.36	−4.02
Potri.001G235300	TIP1;3	−2.30	—	−3.32	−2.42
Potri.006G128000	PIP2;2	−1.48	—	−1.55	—
Potri.006G128200	PIP2;2	−1.83	—	−1.84	—
Potri.008G039600	PIP2;4	—	−1.86	—	−1.93
Potri.019G030900	SIP1;2	—	−2.53	—	—
Potri.009G005400	TIP1;1	—	−1.72	—	—
Potri.T156000	TIP1;1	—	−1.73	—	—
Potri.009G027200	TIP1;3	—	−1.83	−5.18	—
Potri.016G113300	PIP1;4	—	−3.33	—	−1.91

### Effects of canker pathogens on poplar physiology

**Figure 5:**
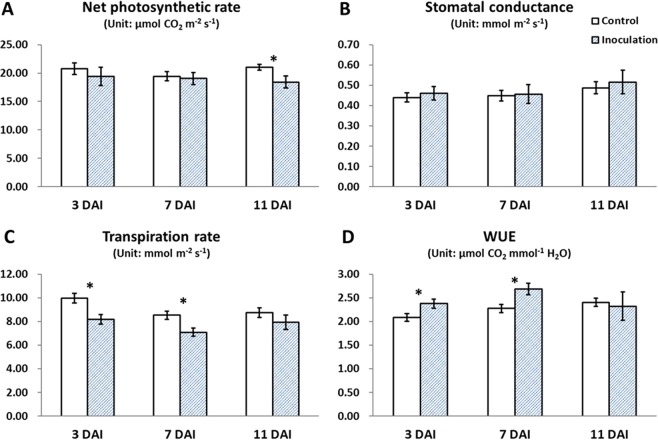
Gas-exchange in poplar trees infected with canker pathogens. (**A**) Net photosynthetic rate. (**B**) Stomatal conductance. (**C**) Transpiration rate. (**D**) WUE, calculated as the formula: net photosynthetic rate divided by transpiration rate. Asterisks indicate treatments that differed significantly from control (ANOVA; *P < 0.05, 6 biological replicates per treatment).

**Figure 6:**
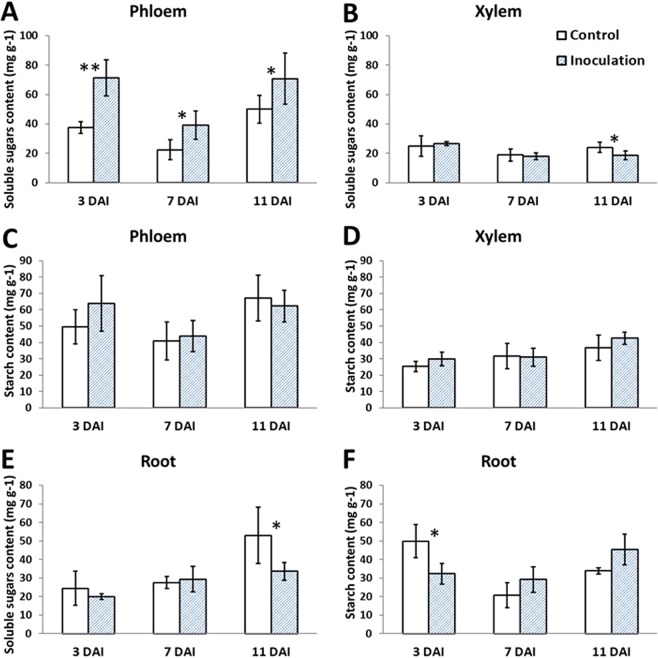
NSC contents in the stem phloem, stem xylem and roots in *Botryosphaeria* infected poplars. showed the content of soluble sugars in stem phloem and xylem, respectively. (**C**,**D**) Showed the starch content in stem phloem and xylem, respectively. (**E**,**F**) Showed the content of soluble sugars and starch in roots, respectively. Asterisks indicate treatments that significantly differed from control (ANOVA; *P < 0.05; **P < 0.01; 6 biological replicates per treatment).

We assessed the gas-exchange characteristics and NSC contents of *Botryosphaeria*-infected poplars. As shown in Fig. 5, pathogen infection reduced the net photosynthetic rate at 11 DAI; pathogen infection also reduced transpiration rate but increased the WUE (water use efficiency) value at 3 and 7 DAI (ANOVA, *P* < 0.05); however, no significant differences in the transpiration rate or WUE were detected at 11 DAI. The NSC content of the stem xylem and phloem tissue was determined at 3, 7, and 11 days after *Botryosphaeria* inoculation. The soluble sugar content of stem phloem samples was significantly increased at 3, 7, and 11 DAI, and decreased in xylem samples at 11 DAI; in contrast, the starch content of phloem and xylem samples was unchanged (ANOVA, *P* < 0.05). In roots, the soluble sugar content was decreased at 11 DAI and the starch content at 3 DAI (ANOVA, *P* < 0.05) (Fig. 6; Supplementary Table S7). The increased sugar content of phloem suggests that pathogen inoculation impeded carbohydrate transport through phloem because pathogen inoculated on the bark. The decreased soluble sugar content of stem xylem and root might associate with the downregulation of genes involved in carbon metabolism at 7 DAI, the obstacles in phloem transport, and the decreased photosynthesis at 11 DAI.

## Discussion

Gene expression profiling of cells and tissues is an important discovery tool in plant science. RNA-seq facilitates genome-wide evaluation of the gene expression profiles of plants under different conditions (*e*.*g*., biotic and abiotic stresses). Under normal conditions, the total number of up- and downregulated DEGs is balanced, but this is disrupted by pathogen infection. However, little research has focused on this issue. For example, malignant transformation of human cells the majority of DEGs were downregulated^27^. Few studies have evaluated this kind of gene expression profiles (the majority of DEGs down- or upregulated) in pathogen inoculated plants.

*Botryosphaeria* and *Valsa* infection were associated with the downregulation of the majority of DEGs in poplar stems at the early stages of canker diseases. This is in agreement with the results of our previous study on the *P*. *trichocarpa–B*. *dothidea* interaction^18^ and miRNA sequencing work^20^. In those studies, 31 of 41 miRNA genes in poplar stem were upregulated at 3, 5, and 7 DAI, 9 miRNA genes were upregulated at one time point at least, and only one miRNA (*ptr-miR1448*) was downregulated at all three time-points^18^. In *P*. *beijingensis*, the overall expression levels of conserved miRNAs were increased by *B*. *dothidea* infection, and these results were validated by RT-qPCR^20^. Considering the negative regulation of target genes by miRNAs, the upregulation of miRNAs indicates that the majority of target genes of miRNAs were inhibited by pathogen attack. Moreover, the downregulated target genes were validated by RT-qPCR^18^. To determine whether the downregulation pattern is the general way of plant respond to fungal pathogens, we re-analyzed the sequencing data of poplar–pathogen interactions^28–30^. The number of down-, upregulated DEGs and their ratio listed in Table 5. However, a similar result was found for the *P*. *tremulodies*/Ston1 (an undescribed *Sphaerulina* species, leaf spot pathogen of poplar) interaction; at 1, 4, and 15 DAI in poplar leaves, the number of downregulated genes was at least 1.36-fold greater than the number of upregulated genes. The majority of the DEGs in the other two *Populus*/*Sphaerulina* interactions and two *Marssonina brunnea*/*Populus* interactions were upregulated^28^. *Sphaerulina musiva* was also canker pathogen which can cause severe stem-girdling cankers in *P*. *trichocarpa* in north American^31^. Transcriptome analysis showed that the majority of DEGs upregulated in the interaction between *P*. *trichocarpa* resistance genotype BESC-801 and *S*. *musiva*, meanwhile, downregulated in *P*. *trichocarpa* susceptible genotype BESC-801/*S*. *musiva* interaction^30^ (Table 5). One transcriptome analysis conducted in our girdling-inoculation system illustrated the downregulation features of poplar DEGs (GBd, GVs and GR vs GC, respectively; 25 DAI; xylem tissue; Xing, Li, *et al*. unpublished data). Considering the difference in pathogen species, resistance and growth time of hosts, treatment time, etc. between our poplar canker diseases and poplar *Sphaerulina* canker disease, the downregulation hypothesis of DEGs in poplar canker diseases is need further investigations.

**Table 5:** DEGs number in fungal pathogen-infected plants.

	Number of up-regulated genes (Nu)	Number of down-regulated genes (Nd)	Differential regulation of DEGs	Number of up-regulated genes (Nu)	Number of down-regulated genes (Nd)	Differential regulation of DEGs	Number of up-regulated genes (Nu)	Number of down-regulated genes (Nd)	Differential regulation of DEGs	Tissues used in RNAseq	Reference
	1 DAI			4 DAI			15 DAI				
*Populus tremulodies*/Ston1	133	323 (70.83)	−2.43*	573	1895 (76.78)	−3.31*	4403	6000 (57.68)	−1.36*	Leaves	^27^
*P*. *deltoides/S*. *musiva*	463	21 (4.33)	22.05**	214	7 (3.17)	30.57**	1775	406 (18.61)	4.37**	Leaves	^27^
*P*. *balsamifera/S*. *populicola*	979	473 (32.58)	2.07**	331	226 (39.86)	1.46*	2060	1230 (37.39)	1.67**	Leaves	^27^
	24 HPI										
*Populus trichocarpa* resistant genotype BESC-22/*Sphaerulina musiva*				3055	1817	1.68*				Stems	^29^
*Populus trichocarpa* susceptible genotype BESC-801/*Sphaerulina musiva*				29	50	−1.72*				Stems	^29^
	6 HPI			36 HPI			96 HPI				
*Marssonina brunnea* f. sp. *Monogermtubi/Populus* sect. *Aigeiros* (Aig)	1453	523 (26.47)	2.78**	511	253 (33.12)	2.02**	1288	319 (20.47)	4.04**	Leaves	^28^
*M*. *brunnea* f. sp. Multigermtubi/*Populus* sect. *Leuce* Duby (Leu)	411	21 (4.86)	19.57**	591	424 (41.77)	1.39**	2,320	1,773 (43.32)	1.30**	Leaves	^28^

Notation: The negative values in this table represent the differential expressed genes (DEGs) are mainly down-regulated in this pathogen-plant interaction, while the positive values represent the DEGs are mainly up-regulated. Asterisks indicate the differential regulation of DEGs are significantly difference (the Chi-square test, the null hypothesis is Nd = Nu; *P < 0.05; **P < 0.01).

Pathogen infection significantly can reduce the net photosynthetic rate and induces carbon starvation in sink tissues^12–15^. In *B*. *dothidea* and *V*. *sordida* inoculated poplars, not only the net photosynthetic rate but also the NSC content of poplar stems decreased at 20, 25 and 30 DAI (Li, Xing, *et al*. 2019, under review), suggesting that pathogen infections decreased carbon reserve by inhibition of photosynthesis. Plant pathogens also influence the carbon reserve by directly depleting NSC reserves, accelerating NSC consumption, and increasing repair costs^16^. *Botryosphaeria dothidea* and *V*. *sordida* are necrotrophic and weakly parasitic organisms, *Botryosphaeria* fungi are even endophytes in healthy plants^32^. The basic survival strategy of all necrotrophic pathogens is to convert living tissue to dead materials^33^, and then pathogens obtained nutrients from these dead materials. For example, a large majority of transcripts encode enzymes (targeting the three most important carbohydrates in hardwood: cellulose, xylan, and pectin) up-regulated in poplar canker pathogen *Mycosphaerella populorum* growing on poplar wood-chip medium^34^. Therefore, we proposed that pathogen *B*. *dothidea* and *V*. *sordida* can derive NSCs directly from living and/or death cells for mycelial growth, particularly at the early stage of canker diseases of poplar. In contrast, tree defense was activated upon pathogen contact with living cells and some carbon-expensive barriers were produced to compartmentalize the pathogen^35^. This process is exemplified by the Norway spruce canker disease: in between the mycelial front and the sapwood, spruce trees have a dry zone where rays and tracheids show embolism and appear enriched with phenolic compounds^35,36^. Therefore, canker pathogens might decrease host carbon reserve through categories pathways.

The carbon and energy consumed in the compartmentalization of pathogens in poplar may decrease the NSC content. The production of disease-resistance proteins requires large amounts of carbon and energy. Then, the upregulation of the majority of disease resistance genes detected in this study implied that this carbon and energy-expensive process started at 7 DAI, or earlier. Host mortality due to pathogen infection is dependent on the balance between carbon reserve and production of disease resistance factors. We report here that during the early stages of infection pathogens can influence the carbon reserve by downregulating the expression of genes related to sugar and starch metabolism in poplar stem tissue prior to the inhibition of photosynthesis (Figs 2, 3 and 6).

Downregulation of carbon metabolism-related genes affects the concentration, distribution, and balance of NSCs in stem xylem and phloem, as well as pathways related to sugar and starch metabolism (such as water transport, disease resistance, *etc*.), facilitating the development of poplar canker disease. *V*. *sordida* fermentation broth was inoculated in poplar stems, the most dramatic inhibition on photosynthesis and stomatal conductivity occurred at 2 DAI, then the inhibition gradually decreased with time, and disappeared at 7 DAI (unpublished data). The change of gene expression was likely induced by the secondary metabolites (such as toxins, secreted proteins, etc.)^37,38^, and so might occur at all stages of canker disease. Therefore, in addition to inhibiting photosynthesis, the two canker pathogens directly modulated carbon metabolism around the inoculation sites.

Drought stress facilitates the development of canker diseases in trees and exacerbates pathogen-induced damage^1–5,39–41^. Timing of drought stress influences host physiology, and host condition influences canker disease susceptibility in eucalypt^39^. Some pathogenic *Botryosphaeriaceae* species have a latent phase, colonizing woody tissues, whereas perennial hosts show no apparent symptoms until conditions become favorable for disease development^42^. In this study, carbon metabolism-related genes were downregulated at 7 DAI, but the photosynthetic rate was inhibited until 11 DAI, thus pathogenesis could be divided into early and late stages according to the gas exchange data. Thus, this study linked the downregulation of carbon metabolism and transport pathway (at or prior to 7 DAI), declination of assimilation (at 11 DAI), and declination of carbon reserve (11 DAI) in a chronological order. In other words, the downregulation of carbon metabolic pathways occurred prior to changes of assimilation rate, even at the early stage of poplar canker diseases.

Results showed that no difference of percent loss of conductivity (PLC) was detected in girdling-*Botryosphaeria* inoculated poplars and girdling control (Li, Xing *et al*. 2019; under review), suggesting that canker pathogens do not induce embolism in stems at middle stage of infection. However, a number of aquaporin-encoding genes were downregulated in stem xylem tissue in poplar. Aquaporins are membrane proteins involved in the repair of drought-induced embolism^43^. In soybean, 24 of 32 PIP-encoding genes were downregulated by *Pseudomonas syringae* infection^44^. Then, we proposed this downregulation pattern was likely to reduce the ability of plants to repair embolism in disease condition. In addition, xylem obstacles caused by gels and tyloses which induced by canker pathogens^45,46^ exacerbated hydraulic failure and carbon starvation in host plants. Finally, the pathogen–drought interaction might exacerbate the pathogen-induced damage^1–5,39–41^. Therefore, the downregulatory pattern of aquaporin-encoding genes suggested that this gene family play crucial roles in plant canker diseases.

In conclusion, genes involved in carbohydrate and starch metabolism were downregulated by infection with the two canker pathogens. Together with carbon depletion due to reduced photosynthesis at the middle stage of canker disease, we also found that the pathogens directly influenced the concentration and distribution of carbohydrates in stem tissues at the early stage of canker disease. Therefore, during pathogen infection the mortality of poplars is associated with carbon starvation. Moreover, via the observed downregulation of aquaporin genes, this study also suggests a potential pathway of tree mortality under drought-pathogen interactions. To our knowledge, this is the first report on the link between carbon starvation and the genome-wide gene expression profile of poplar.

## Materials and Methods

### Plant materials, fungal pathogens, and inoculation

The RNA-seq analysis was undertaken in 2-year-old *Populus beijingensis* clones from cuttings in the greenhouse of the Chinese Academy of Forestry (CAF, Beijing, China) at September, 2015. The poplars were 1.1 to 1.3 m in height, with a mean height of 1.2 m. All of the poplar samples were healthy, without diseases or pests, and were well watered throughout the experiments. Two poplar fungal pathogens, *Botryosphaeria dothidea* isolate CZA and *Valsa sordida* isolate CZC, were cultured on potato dextrose agar (2.0% potato extract, 2.0% dextrose, and 1.5% agar; pH 6.0) for 7 days at 25 °C in the dark. For punch inoculation, saplings were inoculated with four 6-mm-diameter mycelium discs at 30, 35, 40, and 45 cm above the soil and evenly vertically distributed at different heights of the stems. The punch sites were affixed over zones of the stem from which a 6-mm-diameter bark disc had been removed. The controls lacked fungal pathogens. Twelve poplars were used in this experiment, four for *Botryosphaeria dothidea* inoculation (Bdo), four for *Valsa sordida* (Vso) and the other for the control (Ctrl). Seven days after inoculation (DAI), the stems were harvested. To sample tissues for RNA extraction, we separated phloem from xylem, and removed the cambium from the latter by scraping with a razor. To eliminate the influences from mycelium discs and buds in poplar stems, poplar materials (>0.5 cm from their rims) around inoculation sites and buds were removed with a razor and discarded. We established the following six treatment groups: phloem and xylem samples inoculated with *B*. *dothidea*, Bdo_Ph and Bdo_Xy; phloem and xylem samples inoculated with *V*. *sordida*, Vso_Ph and Vso_Xy; and the controls, Ctrl_Ph and Ctrl_Xy. Material was immediately frozen in liquid nitrogen and stored at −80 °C until use.

### RNA extraction, transcriptome sequencing, gene expression analysis, and functional annotation

The modified CTAB method^47^ was used to extract total RNA. RNA was purified and quantified. Only the RNA samples had with a 260/280 ratio of 1.9–2.1, RIN value (RNA integrity number) >7.0, and RNA weight >3 μg were accepted. Moreover, only samples had with qualified phloem and xylem RNA were used to library construct and sequencing. Finally, totally 12 libraries (six groups: Bdo_Ph, Bdo_Xy, Vso_Ph, Vso_Xy, Ctrl_Ph and Ctrl_Xy; 2 replicates per group) were constructed in this study.

Sequencing libraries were generated using 3 μg of RNA per sample and a NEBNext Ultra RNA Library Prep Kit (NEB, Ipswich, MA, USA) following the manufacturer’s recommendations. Briefly, first-strand cDNA was synthesized using random hexamer primers and M-MuLV reverse transcriptase, and second-strand cDNA was synthesized using DNA polymerase I and RNase H. Only RNAs longer than 200 bp were used to construct cDNA libraries. The clean reads were mapped to the *Populus* genome database (Phytozome10, *Populus trichocarpa* v. 3.0) using TopHat2^48^.

Poplar genes were functionally annotated based on the NCBI non-redundant protein (Nr) and non-redundant nucleotide (Nt) (http://www.ncbi.nlm.nih.gov/), Swiss-Prot (http://web.expasy.org/docs/swiss-prot_guideline.html), and Kyoto Encyclopedia of Genes and Genomes (KEGG) (http://www.genome.jp/kegg/kegg2.html) databases^25^. Cufflinks^49^ was used to evaluate transcript expression. Transcript levels are presented as reads per kilobase of exon model per million mapped reads (RPKM) values. Pearson correlation analysis and hierarchical clustering analysis based on the all RPKM data was performed to evaluate the consistency of samples in one group. DEseq. 2^50^ was used in Differentially expressed genes (DEGs) selection. DEGs were identified using the following criteria: log_2_FC (fold change in expression) ≥1 or ≤−1 and false discovery rate (FDR) ≤0.05, or for genes unique to one library, FDR ≤0.05. The cDNA libraries building, RNA sequencing and bioinformatic analysis were conducted by AnnoRoad Gene Technology Corporation (Beijing, China).

### Gas exchange analysis

The gas exchange and NSC content analysis was carried out in 2-year-old *P*. *beijingensis* trees at October 2015. The cultural conditions of poplars and pathogen were same as that in RNA-seq analysis, but only pathogen *B*. *dothidea* isolate CZA was used. Inoculation method and operation was also same as that in RNA-seq analysis. Eighteen poplar saplings were inoculated by pathogen and another eighteen were mock inoculated at the beginning of experiment. The height of poplars was 1.3 ± 0.2 m. All poplars were healthy and were well watered throughout the experiments. Gas exchange was measured at 3, 7, and 11 DAI using a Li-6400 portable gas exchange system (LI-COR, Lincoln, NE, USA). The fifth to sixth fully expanded leaves (from top to base) of six biological replicates per treatment were measured. All measurements were conducted from 10:00 to 11:00 a.m. Photosynthesis was induced under saturating light (1,500 µmol m^−2^ s^−1^) with 370 µmol mol^−1^ CO_2_ surrounding the leaf and a flow rate of 500 µmol air s^−1^. Water use efficiency (WUE) was calculated as net photosynthesis per unit of water transpired (photosynthetic rate/transpiration rate).

### Non-structural carbohydrate concentration assays

NSC contents of six treatments (stem phloem, stem xylem, and root that challenged by *Botryosphaeria dothidea* or not) at 3, 7, and 11 DAI were analyzed. After gas exchange measurement, stem segments were collected from 20-cm regions immediately above the topmost inoculation site. All tissues outside the cambium, referred to as phloem, were carefully peeled from the remaining segment. Next, the cambial tissues were removed, leaving the xylem. Immediately after collection, the samples were heated in an oven at 110 °C for 30 min to stop biological activity. Next, the samples were dried to constant weight at 65 °C for up to 4 h, ground to fine powder, and filtered through a 100-mesh sieve. The concentrations of soluble sugars and starch were estimated using the Plant Soluble Sugars Assay Kit and Starch Assay Kit (Solarbio Life Sciences, Beijing, China) with a microplate reader (Dynamax, USA) at a wavelength of 620 nm. Each treatment had with six biological replicates and each biological replicate had with four technical replicates. Results are presented as mg/g dry weight. Physiological parameters were subjected to analysis of variance (ANOVA) in Microsoft Excel software.

## Supplementary information

Supplementary Indo

Supplementary Dataset 1

Supplementary Dataset 2

Supplementary Dataset 3

## References

[1] Bachi PR, Peterson JL (1989). Enhancement of *Sphaeropsis sapinea* stem invasion of pines by water deficits. Plant Dis..

[2] Blodgett JT, Kruger EL, Stanosz GR (1997). Effects of moderate water stress on disease development by *Sphaeropsis sapinea* on red pine. Phytopathology.

[3] Madar Z, Solel Z, Kimchi M (1989). Effect of water stress in cypress on the development of cankers caused by *Diplodia pinea* f.sp. *cupressi* and *Seiridium cardinale*. Plant Dis..

[4] Maxwell DL, Kruger E, Stanosz GR (1997). Effects of water stress on colonization of poplar stems and excised leaf disks by *Septoria musiva*. Phytopathology.

[5] Paoletti E, Danti R, Strati S (2001). Pre- and post-inoculation water stress affects *Sphaeropsis sapinea* canker length in *Pinus halepensis* seedlings. Forest Pathol..

[6] SFA. The occurrence of the main forestry diseases and pests at 2010 and the prediction at 2011 in China. Forest Protection Bulletin **1** (2011).

[7] Liang, J., Yan, D. & Zhang, X. Cytospora canker of China. In: *Major forest diseases and insect pests in China*, 120–139. China Forestry Publishing House, Beijing (2003).

[8] Lv, Q., Liu, H. & Zhang, X. Poplar canker. In: *Major forest diseases and insect pests in China*, 95–119. China Forestry Publishing House, Beijing (2003).

[9] McDowell N (2008). Mechanisms of plant survival and mortality during drought: why do some plants survive while others succumb to drought?. New Phytol..

[10] Martinez-Vilalta J (2014). Carbon storage in trees: pathogens have their say. Tree Physiol..

[11] Aguade D, Poyatos R, Gómez M, Oliva J, Martínez-Vilalta J (2015). The role of defoliation and root rot pathogen infection in driving the mode of drought-related physiological decline in Scots pine (*Pinus sylvestris* L.). Tree Physiol..

[12] Rohrs-Richey JK, Mulder CP, Winton LM, Stanosz G (2011). Physiological performance of an Alaskan shrub (*Alnus fruticosa*) in response to disease (*Valsa melanodiscus*) and water stress. New Phytol..

[13] da Silva AC (2018). Eucalypt plants are physiologically and metabolically affected by infection with Ceratocystis fimbriata. Plant Physiol Biochem..

[14] Gortari F, Guiamet JJ, Graciano C (2018). Plant-pathogen interactions: leaf physiology alterations in poplars infected with rust (*Melampsora medusae*). Tree Physiol..

[15] Plichta R, Urban J, Gebauer R, Dvořák M, Ďurkovič J (2006). Long-term impact of *Ophiostoma novo-ulmi* on leaf traits and transpiration of branches in the Dutch elm hybrid ‘Dodoens’. Tree Physiol..

[16] Oliva J, Stenlid J, Martínez-Vilalta J (2014). The effect of fungal pathogens on the water and carbon economy of trees: implications for drought-induced mortality. New Phytol..

[17] Major IT, Nicole MC, Duplessis S, Séguin A (2010). Photosynthetic and respiratory changes in leaves of poplar elicited by rust infection. Photosynth Res..

[18] Zhao J (2012). Involvement of microRNA-mediated gene expression regulation in the pathological development of stem canker disease in *Populus trichocarpa*. PLoS One.

[19] Wu Q, Chen M, Zhou H, Zhou X, Wang Y (2015). Metabolite profiles of *Populus* in response to pathogen stress. Biochem Biophys Res Commun..

[20] Chen L (2012). Genome-wide profiling of novel and conserved *Populus* microRNAs involved in pathogen stress response by deep sequencing. Planta.

[21] Liao W (2014). Identification of glutathione S-transferase genes responding to pathogen infestation in *Populus tomentosa*. Funct Integr Genomics..

[22] Zhao J (2017). Genome-wide constitutively expressed gene analysis and new reference gene selection based on transcriptome data: a case study from poplar/canker disease interaction. Front Plant Sci..

[23] Yadeta K, Thomma B (2013). The xylem as battleground for plant hosts and vascular wilt pathogens. Front Plant Sci..

[24] Brunner AM, Busov VB, Strauss SH (2004). Poplar genome sequence: functional genomics in an ecologically dominant plant species. Trends Plant Sci..

[25] Kanehisa M, Goto S (2000). KEGG: Kyoto Encyclopedia of Genes and Genomes. Nucleic Acids Res..

[26] Chen F (2018). The WRKY transcription factor family in model plants and crops. Crit Rev Plant Sci..

[27] Danielsson F (2013). Majority of differentially expressed genes are down-regulated during malignant transformation in a four-stage model. PNAS.

[28] Foster AJ, Pelletier G, Tanguay P, Séguin A (2015). Transcriptome analysis of poplar during leaf spot infection with *Sphaerulina* spp. PLoS One.

[29] Zhang Y, Tian L, Yan D, He W (2018). Genome-wide transcriptome analysis reveals the comprehensive response of two susceptible poplar sections to *Marssonina brunnea* infection. Genes (Basel).

[30] Muchero W (2018). Association mapping, transcriptomics, and transient expression identify candidate genes mediating plant-pathogen interactions in a tree. PNAS.

[31] Feau N, Mottet MJ, Périnet P, Hamelin RC, Bernier L (2010). Recent advances related to poplar leaf spot and canker caused by *Septoria musiva*. Can J Plant Pathol..

[32] Slippers B, Wingfield MJ (2007). *Botryosphaeriaceae* as endophytes and latent pathogens of woody plants: diversity, ecology and impact. Fungal Biol Rev..

[33] Oliver RP, Solomon PS (2010). New developments in pathogenicity and virulence of necrotrophs. Curr Opin Plant Biol..

[34] Dhillon B (2015). Horizontal gene transfer and gene dosage drives adaptation to wood colonization in a tree pathogen. PNAS.

[35] Oliva J, Camarero JJ, Stenlid J (2012). Understanding the role of sapwood loss and reaction zone formation on radial growth of Norway spruce (*Picea abies*) trees decayed by *Heterobasidion annosum s*.*l*. Forest Ecology and Management.

[36] Johansson M, Stenlid J (1985). Infection of roots of Norway spruce (*Picea abies*) by *Heterobasidion annosum*. Eur. J. For. Pathol..

[37] Doehlemann, G., Ökmen, B., Zhu, W. & Sharon, A. Plant Pathogenic Fungi. *Microbiol Spectr*. **5**(**1**), 10.1128/microbiolspec.FUNK-0023-2016 (2017). 10.1128/microbiolspec.funk-0023-2016 PMC11687436 28155813

[38] Stempien E (2018). Secreted proteins produced by fungi associated with *Botryosphaeria* dieback trigger distinct defense responses in *Vitis vinifera* and *Vitis rupestris* cells. Protoplasma.

[39] Hossain M (2019). Tree host-pathogen interactions as influenced by drought timing: linking physiological performance, biochemical defence and disease severity. Tree Physiol..

[40] Desprez-Loustau M-L (2007). Simulating the effects of a climate-change scenario on the geographical range and activity of forest-pathogenic fungi. Can J Plant Pathol..

[41] La Porta N (2008). Forest pathogens with higher damage potential due to climate change in Europe. Can J Plant Pathol..

[42] Marsberg A (2017). *Botryosphaeria dothidea*: a latent pathogen of global importance to woody plant health. Mol Plant Pathol..

[43] Afzal Z, Howton TC, Sun Y, Mukhtar MS (2014). The Roles of aquaporins in plant stress responses. J Dev Biol..

[44] Zou J (2005). Expression profiling soybean response to *Pseudomonas syringae* reveals new defense-related genes and rapid HR-specific downregulation of photosynthesis. Mol Plant Microbe Interact..

[45] Biggs AR (1983). Histology of cankers on Populus caused by *Cytospora chrysosperma*. Can J Bot..

[46] Milholland RD (1970). Histology of Botryosphaeria canker of susceptible and resistant Highbush Blueberry. Phytopathology.

[47] Chang S (1993). A simple and efficient method for isolating RNA from pine trees. Plant Mol Biol Report..

[48] Kim D (2013). TopHat2: accurate alignment of transcriptomes in the presence of insertions, deletions and gene fusions. Genome Biol..

[49] Trapnell C (2010). Transcript assembly and quantification by RNA-Seq reveals unannotated transcripts and isoform switching during cell differentiation. Nat Biotechnology.

[50] Love MI, Huber W, Anders S (2014). Moderated estimation of fold change and dispersion for RNA-seq data with DESeq2. Genome Biology.

