# Supplementary material for: Fungal canker pathogens trigger carbon starvation by inhibiting carbon metabolism in poplar stems
Source: Sci Rep. 2019 Jul 12;9:10111. doi: 10.1038/s41598-019-46635-5 (PMC6626041; doi:10.1038/s41598-019-46635-5)
Supplement: Supplementary file 1 — Supplementary Indo [file 41598_2019_46635_MOESM1_ESM.doc]

Fungal canker pathogens trigger carbon starvation by inhibiting carbon metabolism in poplar stems

Ping Li^1,*^, Wenxin Liu^1,*^, Yinan Zhang^1^, Junchao Xing^1^, Jinxin Li^1^, Jinxia Feng^1^, Xiaohua Su^2,†^, Jiaping Zhao^1,†^

^1^State Key Laboratory of Tree Genetics and Breeding, Forestry Institute of New Technology, Chinese Academy of Forestry, Beijing, China

^2^State Key Laboratory of Tree Genetics and Breeding, Institute of Forestry, Chinese Academy of Forestry, Beijing, China

* These authors contributed equally to this work.

† Corresponding author:

Jiaping Zhao, Email: zhaojiaping@caf.ac.cn; Tel: 86-1-62889474.

Xiaohua Su, Email: suxh@caf.ac.cn.

**Supplementary materials**

Table S1. Pearson correlation analysis of RNA sequencing data.

| Sample | A11M | A11P | C10M | C10P | CK10M | CK10P |
| --- | --- | --- | --- | --- | --- | --- |
| A9M | 0.8821 |  |  |  |  |  |
| A9P | 0.5136 | 0.9987 |  |  |  |  |
| C9M | 0.8931 | 0.3592 | 0.6953 |  |  |  |
| C9P | 0.5092 | 0.9978 | 0.1777 | 0.9986 |  |  |
| CK9M | 0.8597 | 0.2122 | 0.7365 | 0.2080 | 0.9009 |  |
| CK9P | 0.4711 | 0.9990 | 0.1304 | 0.9974 | 0.1378 | 0.9973 |

Table S2. Differentially expressed transcription factors genes in canker pathogens inoculated poplars.

| Treatments | Bdo_Xy (Gene_ID log_2_FC) | Bdo_Ph (Gene_ID log_2_FC) | Vso_Xy (Gene_ID log_2_FC) | Vso_Ph (Gene_ID log_2_FC) |
| --- | --- | --- | --- | --- |
| Transcription factors | Potri.001G080900 -4.60  Potri.002G040600 1.73  Potri.001G139900 -2.34  Potri.001G235500 1.14  Potri.010G208600 -2.37  Potri.011G041600 -5.14  Potri.012G055700 -3.56  Potri.013G067000 -3.07  Potri.015G046300 -2.50  Potri.003G074400 1.53  Potri.003G144300 -1.41  Potri.003G147300 -4.75  Potri.001G248800 1.06  Potri.006G074600 -1.75  Potri.009G064700 -1.22  Potri.011G129500 1.70  Potri.012G055600 -2.01  Potri.014G148900 2.18  Potri.015G041100 -3.40  Potri.015G046200 -3.77  Potri.015G069000 1.74  Potri.019G050900 -3.03  Potri.001G267300 -3.16  Potri.001G270000 1.09  Potri.001G410600 1.23  Potri.003G051600 1.15  Potri.004G156000 1.21  Potri.005G039800 -1.62  Potri.005G186400 -2.57  Potri.006G177000 -1.93  Potri.007G066300 -2.88  Potri.007G093900 -2.11  Potri.008G070800 1.28  Potri.008G142700 1.79  Potri.008G191800 1.71  Potri.009G053900 -3.36  Potri.009G081400 -2.07  Potri.011G030900 1.36  Potri.015G033600 -1.96  Potri.016G112300 1.14  Potri.017G017600 -2.94 | Potri.002G113700 -1.10  Potri.006G251800 -1.35  Potri.008G122100 -4.55  Potri.010G149900 -2.30  Potri.010G208600 -2.69  Potri.011G041600 -1.11  Potri.012G055700 -3.33  Potri.013G067000 -2.15  Potri.015G046300 -1.53  Potri.017G015800 -1.16  Potri.019G112000 1.38 | Potri.001G080900 -6.01  Potri.002G040600 1.54  Potri.001G063000 -5.84  Potri.010G149900 -4.32  Potri.001G083500 -2.11  Potri.011G041600 -5.42  Potri.012G055700 -2.18  Potri.013G067000 -5.69  Potri.004G046300 1.98  Potri.003G074400 2.27  Potri.001G410600 2.95  Potri.003G147300 -6.57  Potri.003G149700 -5.45  Potri.001G375800 3.20  Potri.009G064700 -1.72  Potri.011G129500 3.61  Potri.012G055600 -3.93  Potri.014G148900 2.36  Potri.015G041100 -4.63  Potri.015G046200 -4.23  Potri.015G069000 1.94  Potri.019G050900 -7.29  Potri.001G086700 -5.86  Potri.002G057200 -6.17  Potri.002G122600 1.75  Potri.002G152200 -3.69  Potri.004G029100 1.54  Potri.006G058800 -6.34  Potri.006G074600 -3.15  Potri.006G074900 2.50  Potri.006G234200 -4.54  Potri.007G098800 2.14  Potri.008G101400 -2.31  Potri.008G142700 2.75  Potri.009G125400 1.54  Potri.013G110700 1.80  Potri.013G129800 -1.99  Potri.014G111200 -1.79  Potri.015G074500 -6.04  Potri.016G049200 -5.09  Potri.016G099200 -4.52 | Potri.001G083500 -4.01  Potri.001G219100 -1.62  Potri.008G122100 -6.61  Potri.010G149900 -3.94  Potri.010G208600 -1.83  Potri.011G041600 -1.09  Potri.012G055700 -3.78  Potri.002G122600 1.83  Potri.002G128900 2.55  Potri.003G189700 1.02  Potri.003G144300 -1.53  Potri.003G147300 -5.71  Potri.003G149700 -1.73  Potri.006G074600 -2.50  Potri.009G064700 -1.73  Potri.006G205100 -1.30  Potri.012G055600 -1.14  Potri.014G148900 1.26  Potri.007G048900 1.17  Potri.009G035000 -1.31  Potri.014G022500 1.52  Potri.014G103700 -1.29  Potri.015G075800 -2.02  Potri.015G134300 -3.38  Potri.016G050500 1.18  Potri.016G072300 -2.01  Potri.T044100 1.87 |
| Regulation | 16 Up; 24 Down | 10 Down; 1 Up | 25 Down; 15 Up; | 18 Down; 8 Up; |

Notation: The gene ID with the underline represents the gene co-expressed at least two treatments.

Table S3. Number of DE genes enriched in different metabolic pathways in poplar under the attack of canker pathogens.

| Metabolism pathway | Bdo_Xy | | Vso_Ph | | Vso_Xy | |
| --- | --- | --- | --- | --- | --- | --- |
|  | Number of down-regulated genes | Number of up-regulated genes | Number of down-regulated genes (Nd) | Number of up-regulated genes (Nu) | Number of down-regulated genes (Nd) | Number of up-regulated genes (Nu) |
| Biosynthesis of secondary metabolites | 114 | 19 | 40 | 5 | 59 | 1 |
| Metabolic pathways | 176 | 34 |  |  | 61 | 19 |
| Phenylpropanoid biosynthesis | 22 | 7 |  |  |  |  |
| Starch and sucrose metabolism | 33 | 7 |  |  | 8 | 2 |
| Amino sugar and nucleotide sugar metabolism | 26 | 2 |  |  |  |  |
| Biosynthesis of amino acids | 35 | 3 |  |  | 15 | 3 |
| Plant hormone signal transduction | 22 | 17 | 12 | 6 | 10 | 5 |
| Plant-pathogen interaction |  |  | 7 | 11 |  |  |

Notation: No significantly KEGG enrichment result derived from the transcriptome data of Bdo_Ph vs Ctrl_Ph.

**Table S4. DE genes involved in biosynthesis of** **secondary metabolites pathway and phenylpropanoid biosynthesis pathway.**

| Description (Uniprot) | Bdo_Xy | | Vso_Xy | |
| --- | --- | --- | --- | --- |
|  | Gene ID | Log2FC | Gene ID | Log2FC |
| 1-aminocyclopropane-1-carboxylate oxidase | Potri.006G151600 | -3.12 | Potri.006G151600 | -2.87 |
|  | Potri.011G020900 | -5.48 | Potri.011G020900 | -5.46 |
|  | Potri.014G159000 | -1.73 | Potri.002G224100 | -2.39 |
|  | Potri.002G078600 | -4.37 | Potri.010G073200 | -2.95 |
|  | Potri.008G165400 | -3.23 | Potri.005G222300 | -3.34 |
|  |  |  | Potri.003G132300 | -4.51 |
|  |  |  | Potri.001G099400 | 2.48 |
| 1-aminocyclopropane-1-carboxylate synthase | Potri.007G007800 | -2.47 | - | - |
|  | Potri.003G132300 | -4.04 |  |  |
|  | Potri.002G163700 | -3.41 |  |  |
| 1-epimerase | - | - | Potri.017G080200 | -4.64 |
| 2,3-bisphosphoglycerate-dependent phosphoglycerate mutase | Potri.002G093300 | -2.19 | - | - |
| 2,3-bisphosphoglycerate-independent phosphoglycerate mutase | Potri.006G113300 | -1.09 | - | - |
| 2-oxoisovalerate dehydrogenase | Potri.005G185400 | -2.83 | - | - |
| 3-isopropylmalate dehydratase | Potri.008G172500 | -3.19 | - | - |
| 3-ketoacyl-CoA synthase | Potri.002G178000 | -4.03 | Potri.002G178000 | -5.66 |
|  | Potri.008G160000 | -3.51 | Potri.008G160000 | -3.49 |
|  | Potri.010G125300 | -3.99 | Potri.010G125300 | -4.50 |
|  | Potri.001G234500 | -2.50 |  |  |
|  | Potri.009G116700 | -1.78 |  |  |
|  | Potri.010G079300 | -3.32 |  |  |
| 3-methyl-2-oxobutanoate hydroxymethyltransferase | Potri.014G090500 | -1.80 | Potri.014G090500 | -2.10 |
| 5-methyltetrahydropteroyltriglutamate--homocysteine methyltransferase | Potri.004G190900 | -1.34 | - |  |
|  | Potri.T070100 | -1.45 |  |  |
|  | Potri.013G061800 | -2.09 |  |  |
|  | Potri.019G038200 | -1.53 |  |  |
| 6-phosphogluconate dehydrogenase | Potri.017G106900 | -1.40 | - | - |
| 9-cis-epoxycarotenoid dioxygenase | - | - | Potri.001G393800 | -2.09 |
|  |  |  | Potri.003G176300 | 2.90 |
| Abscisic-aldehyde oxidase | Potri.004G191300 | 1.27 | - |  |
| Acidic endochitinase | - | - | Potri.012G033900 | 2.05 |
|  |  |  | Potri.015G024000 | -5.40 |
|  |  |  | Potri.015G023900 | -4.27 |
| Acidic mammalian chitinase | Potri.018G112000 | 2.35 | - | - |
|  | Potri.018G112100 | -1.79 |  |  |
| Adenylate kinase | Potri.015G092800 | -1.89 | - | - |
| Alcohol dehydrogenase | Potri.004G067000 | -1.67 | Potri.002G013400 | -4.33 |
| Aldehyde dehydrogenase * | Potri.015G074100 | 1.02 | - | - |
|  | Potri.018G075000 | -2.29 |  |  |
|  | Potri.001G167100 | 1.06 |  |  |
| Aldose 1-epimerase | Potri.004G129700 | -1.41 | - | - |
|  | Potri.017G080200 | -3.64 |  |  |
| Anthocyanidin reductase | Potri.004G030700 | -3.55 | Potri.004G030700 | -5.26 |
| Arogenate dehydratase/prephenate dehydratase | Potri.004G013400 | -1.38 | Potri.004G013400 | -1.65 |
|  | Potri.011G004700 | -1.43 |  |  |
|  | Potri.009G148800 | -2.03 |  |  |
| Arogenate dehydrogenase | Potri.008G074500 | -1.62 | Potri.008G074500 | -3.50 |
| Asparagine synthetase | Potri.009G072900 | -3.94 | Potri.009G072900 | -3.51 |
|  | Potri.001G278400 | -5.11 | Potri.001G278400 | -4.74 |
| Aspartate aminotransferase | Potri.018G082500 | -1.06 |  |  |
|  | Potri.006G107100 | -1.17 |  |  |
| Aspartate-semialdehyde dehydrogenase | Potri.008G135700 | -1.02 |  |  |
| Aspartic proteinase nepenthesin-2 |  |  | Potri.018G014600 | -2.14 |
| Aspartic proteinase-like protein |  |  | Potri.002G092100 | -2.83 |
| Aspartokinase | Potri.002G236800 | -1.49 | Potri.002G236800 | -1.78 |
| ATP-citrate synthase | Potri.010G042700 | -2.17 |  |  |
|  | Potri.008G105300 | -1.26 |  |  |
| Beta-glucosidase * | Potri.008G094200 | -2.55 | Potri.008G094200 | -2.58 |
|  | Potri.004G109200 | Up |  |  |
|  | Potri.001G222900 | -2.41 |  |  |
|  | Potri.015G041300 | -2.14 |  |  |
|  | Potri.004G019300 | 1.61 |  |  |
|  | Potri.004G019400 | 1.96 |  |  |
|  | Potri.004G019700 | 1.90 |  |  |
|  | Potri.004G019800 | 1.24 |  |  |
|  | Potri.013G055600 | -1.06 |  |  |
|  | Potri.009G153900 | -2.00 |  |  |
| Beta-hexosaminidase | Potri.008G079400 | -1.60 | - | - |
| Bifunctional 3-dehydroquinate dehydratase/shikimate dehydrogenase | Potri.014G135500 | -2.41 | - | - |
|  | Potri.005G043400 | -1.55 |  |  |
|  | Potri.T107000 | -2.12 |  |  |
| Bifunctional dihydroflavonol 4-reductase/flavanone 4-reductase | Potri.002G033600 | -3.46 | Potri.002G033600 | -4.73 |
| Bifunctional dTDP-4-dehydrorhamnose 3,5-epimerase/dTDP-4-dehydrorhamnose reductase | Potri.001G112000 | -1.24 | - | - |
|  | Potri.003G120000 | -1.22 |  |  |
| Caffeoyl-CoA O-methyltransferase * | Potri.009G099800 | -1.07 | - | - |
| Chitotriosidase | Potri.006G188400 | -5.42 | - | - |
|  | Potri.006G188300 | -3.55 |  |  |
| Cinnamoyl-CoA reductase * | Potri.018G100500 | -2.81 | Potri.018G100500 | -2.81 |
|  |  |  | Potri.006G178700 | -4.66 |
|  |  |  | Potri.001G046400 | -2.88 |
|  |  |  | Potri.003G093700 | 3.32 |
|  |  |  | Potri.001G140700 | -6.17 |
|  |  |  | Potri.004G105000 | -4.87 |
|  |  |  | Potri.017G110500 | -5.15 |
| Cycloartenol synthase | Potri.006G079100 | -1.46 | - | - |
| Cycloartenol-C-24-methyltransferase | Potri.001G263700 | -1.68 | - | - |
| Cytochrome P450 * | Potri.009G065000 | -1.10 | Potri.001G270900 | -5.72 |
|  | Potri.018G062100 | 1.54 |  |  |
|  | Potri.004G017700 | -1.16 | Potri.008G205200 | -3.26 |
|  | Potri.006G141400 | -3.07 | Potri.003G007000 | -2.97 |
|  | Potri.016G137400 | 3.22 |  |  |
|  | Potri.015G086000 | 1.00 |  |  |
|  | Potri.003G066400 | -2.16 | Potri.002G025400 | 2.03 |
|  | Potri.018G051300 | -2.71 | Potri.002G026100 | 1.88 |
|  | Potri.001G167800 | -2.07 | Potri.002G025300 | 1.92 |
|  | Potri.016G137600 | 2.59 | Potri.018G146100 | -4.92 |
|  | Potri.002G263800 | 2.26 |  |  |
|  | Potri.005G034500 | -2.88 |  |  |
|  | Potri.002G150300 | -4.25 |  |  |
|  | Potri.014G020600 | -2.76 | Potri.001G270800 | -2.81 |
|  | Potri.014G020700 | -2.83 |  |  |
|  | Potri.001G334700 | 1.15 |  |  |
|  | Potri.014G037800 | 1.72 | Potri.011G117600 | -2.40 |
|  | Potri.013G125300 | -4.73 | Potri.013G125300 | -4.56 |
|  | Potri.004G106600 | -4.26 | Potri.004G106600 | -5.49 |
|  | Potri.009G043700 | -5.69 | Potri.009G043700 | -4.09 |
|  | Potri.001G249700 | -4.47 | Potri.001G249700 | -4.44 |
|  | Potri.014G085800 | -3.90 |  |  |
|  | Potri.007G072100 | -3.67 | Potri.007G072100 | -3.80 |
|  | Potri.005G092200 | -5.31 | Potri.005G092200 | -5.66 |
|  | Potri.005G064400 | -3.60 | Potri.003G066400 | -2.08 |
|  | Potri.001G270700 | -2.53 |  |  |
|  | Potri.009G064800 | 1.93 | Potri.009G064800 | 1.83 |
|  | Potri.015G006100 | 1.29 |  |  |
|  | Potri.008G067500 | 1.22 |  |  |
|  | Potri.016G031800 | 1.79 |  |  |
|  | Potri.016G031700 | 1.93 |  |  |
|  | Potri.016G031100 | -3.17 | Potri.016G031100 | -3.58 |
| Cytokinin hydroxylase | - |  | Potri.004G100400 | 3.22 |
| Dihydroflavonol-4-reductase | Potri.003G138400 | -2.63 | - | - |
| Diphosphomevalonate decarboxylase | Potri.010G237900 | -1.11 | - | - |
| Endonuclease | Potri.004G041900 | 1.28 | - | - |
| Enolase | Potri.015G131100 | -1.17 | - | - |
|  | Potri.006G116800 | -1.07 |  |  |
|  | Potri.012G057500 | -1.14 |  |  |
| Enoyl-[acyl-carrier-protein] reductase | Potri.016G046700 | -2.43 | - | - |
| Ent-kaurene oxidase | Potri.002G129700 | 1.39 | Potri.002G129700 | 1.62 |
| Flavonol synthase/flavanone 3-hydroxylase | - | - | Potri.011G164200 | -4.16 |
|  |  |  | Potri.011G150400 | -4.17 |
| Fructose-bisphosphate aldolase | Potri.006G165700 | -1.15 | Potri.006G165700 | -1.44 |
|  | Potri.004G162400 | 1.33 |  |  |
|  | Potri.007G015500 | 1.70 |  |  |
|  | Potri.018G090100 | -1.47 |  |  |
| Gibberellin 20 oxidase | - | - | Potri.015G134600 | 3.23 |
| Glucose-1-phosphate adenylyltransferase | Potri.005G229700 | 1.06 | - |  |
|  | Potri.014G171800 | 1.02 |  |  |
| Glucose-6-phosphate 1-dehydrogenase | Potri.005G006100 | 1.42 | - | - |
|  | Potri.001G337400 | -1.11 |  |  |
| Glucose-6-phosphate/phosphate translocator | Potri.004G019900 | 2.46 | - | - |
| Glutamate decarboxylase | Potri.T059200 | -1.86 | Potri.T059200 | -1.83 |
|  | Potri.010G100500 | -2.50 | Potri.012G039000 | 1.54 |
| Glutamate dehydrogenase | Potri.012G113500 | -2.38 | - | - |
| Glutamate synthase | Potri.015G017500 | -1.16 | - | - |
| Glutamine--fructose-6-phosphate aminotransferase | Potri.019G054500 | -1.10 | - | - |
| Glutamyl-tRNA reductase | - | - | Potri.009G080600 | 2.00 |
| Glyceraldehyde-3-phosphate dehydrogenase | Potri.010G055400 | -2.25 | Potri.010G055400 | -3.91 |
|  | Potri.008G179300 | -1.70 |  |  |
|  | Potri.005G254100 | 1.08 |  |  |
|  | Potri.008G083900 | -1.59 |  |  |
| Heterodimeric geranylgeranyl pyrophosphate synthase | Potri.015G043400 | -1.59 | Potri.015G043400 | -2.75 |
| Homocysteine S-methyltransferase | Potri.005G213100 | -1.07 | Potri.008G155900 | 2.82 |
| Hydrolases family 18 | - | - | Potri.006G188400 | -5.45 |
|  |  |  | Potri.018G112000 | 3.03 |
| Hydroxymethylglutaryl-CoA synthase | Potri.003G120300 | -1.30 | Potri.003G120300 | -1.94 |
| Kinases N terminal domain | Potri.001G111700 | -1.31 | Potri.010G237900 | -1.55 |
| Malate dehydrogenase | Potri.002G141700 | -3.28 | - | - |
| Mannose-1-phosphate guanylyltransferase | Potri.008G060100 | -1.40 | - | - |
| NADH--cytochrome b5 reductase | Potri.013G067300 | -1.15 | - | - |
| Peroxidase * | Potri.004G144600 | 1.66 | Potri.004G144600 | 3.03 |
|  | Potri.010G134500 | -5.10 | Potri.010G134500 | -5.35 |
|  | Potri.005G135300 | -2.99 | Potri.006G069600 | 2.43 |
|  | Potri.008G110600 | -5.59 | Potri.016G058200 | -4.30 |
|  | Potri.005G195700 | -3.54 | Potri.005G195700 | -4.30 |
|  | Potri.003G214900 | -5.52 | Potri.003G214900 | -4.94 |
|  | Potri.005G072800 | 1.17 | Potri.017G038100 | 1.62 |
|  | Potri.006G129900 | 1.44 | Potri.006G129900 | 2.38 |
|  | Potri.017G037900 | -3.03 | Potri.017G037900 | 2.05 |
|  | Potri.001G351000 | -1.36 | Potri.013G083600 | -5.85 |
|  | Potri.013G156500 | -3.30 | Potri.013G156500 | -3.67 |
|  | Potri.004G134800 | 1.66 | Potri.004G134800 | 3.08 |
|  | Potri.014G143200 | -3.45 | Potri.014G143200 | -4.49 |
|  | Potri.002G018000 | -3.19 | Potri.017G064100 | -7.05 |
|  | Potri.007G053400 | -3.29 | Potri.007G053400 | -3.72 |
|  | Potri.001G013000 | -4.34 | Potri.001G013000 | -2.88 |
|  | Potri.001G011200 | -4.83 | Potri.001G011200 | -3.41 |
|  | Potri.001G011000 | -5.62 | Potri.001G011000 | -4.16 |
|  | Potri.001G011300 | -4.63 | Potri.001G011300 | -4.62 |
|  | Potri.016G125000 | -5.30 | Potri.016G125000 | -3.79 |
| Peroxisomal (S)-2-hydroxy-acid oxidase | Potri.003G069300 | 1.16 |  | - |
| Phosphatase superfamily |  |  | Potri.002G093300 | -2.27 |
| Phospho-2-dehydro-3-deoxyheptonate aldolase | Potri.005G073300 | -2.50 | Potri.005G073300 | -2.50 |
|  | Potri.005G162800 | -1.45 |  |  |
|  | Potri.002G099200 | -1.52 |  |  |
|  | Potri.001G150500 | -1.11 |  |  |
| Phytoene synthase | Potri.017G138900 | -2.66 | Potri.017G138900 | -2.35 |
|  | Potri.005G205800 | 1.60 | Potri.005G205800 | 1.57 |
|  | Potri.002G056800 | 1.43 | Potri.002G056800 | 1.56 |
| Polygalacturonate 4-alpha-galacturonosyltransferase | Potri.014G073800 | -1.10 |  |  |
| Polyphenol oxidase | Potri.001G388900 | -4.79 | Potri.001G388900 | -5.26 |
|  | Potri.011G047300 | -6.34 | Potri.011G047300 | -7.05 |
|  | Potri.011G047200 | -2.10 |  |  |
|  | Potri.001G387900 | -2.37 |  |  |
| Probable 4-coumarate--CoA ligase * | Potri.T071600 | -4.16 |  |  |
| Probable aminotransferase | Potri.017G014100 | -1.42 |  |  |
| Probable carotenoid cleavage dioxygenase |  |  | Potri.005G069100 | 1.98 |
| Probable cinnamyl alcohol dehydrogenase * | Potri.002G018300 | -1.91 | Potri.002G018300 | -2.71 |
| Probable galacturonosyltransferase | Potri.001G416800 | -2.21 | Potri.016G001700 | 1.45 |
|  | Potri.011G132600 | -3.30 | Potri.007G031700 | 2.14 |
|  | Potri.017G106800 | -1.22 | Potri.010G129400 | 2.76 |
|  | Potri.014G040300 | -1.05 | Potri.002G200200 | 2.24 |
| Probable galacturonosyltransferase | Potri.014G125000 | -1.55 | Potri.008G192600 | 3.46 |
| Probable malate dehydrogenase | Potri.007G009100 | -1.31 |  |  |
| Probable ribose-5-phosphate isomerase | Potri.010G115300 | -1.60 | Potri.008G127600 | 2.74 |
| Proline dehydrogenase | Potri.004G106400 | 1.84 |  |  |
| Prolycopene isomerase | Potri.006G199600 | 1.11 |  |  |
| Pyruvate dehydrogenase | Potri.010G038400 | -1.19 |  |  |
| S-adenosylmethionine synthase | Potri.002G189000 | -1.47 |  |  |
|  | Potri.013G004100 | -1.25 |  |  |
|  | Potri.008G099300 | -1.46 |  |  |
|  | Potri.010G153500 | -1.64 |  |  |
|  | Potri.006G123200 | -1.46 |  |  |
| Serine decarboxylase | Potri.005G190500 | -1.25 |  |  |
| Serine hydroxymethyltransferase | Potri.001G320400 | -1.25 |  |  |
| Shikimate O-hydroxycinnamoyltransferase * | Potri.018G105500 | -3.51 |  |  |
|  | Potri.001G042900 | -1.27 |  |  |
|  | Potri.001G128100 | 3.11 |  |  |
| Shikimate dehydrogenase |  |  | Potri.014G135500 | -2.99 |
|  |  |  | Potri.T107000 | -3.10 |
| Sterol 24-C-methyltransferase | Potri.008G181800 | Up | - | - |
| Tetraketide alpha-pyrone reductase * | Potri.006G178700 | -3.33 |  |  |
|  | Potri.001G140700 | -3.96 |  |  |
| Threonine dehydratase biosynthetic, chloroplastic | Potri.006G118400 | -1.14 | - | - |
| Transketolase, chloroplastic | Potri.014G068200 | -1.36 | - | - |
| UDP-arabinose 4-epimerase | Potri.001G459700 | -1.34 | Potri.001G459700 | -1.56 |
| UDP-glucose 6-dehydrogenase | Potri.004G118600 | -2.47 | - | - |
|  | Potri.017G092000 | -1.32 |  |  |
|  | Potri.008G094300 | -2.87 |  |  |
| UDP-N-acetylglucosamine diphosphorylase | Potri.003G074700 | -1.46 | - | - |
|  | Potri.001G159700 | -1.02 |  |  |
| Xanthoxin dehydrogenase | Potri.001G024300 | 1.02 | - | - |

Notation：The asterisk (*) indicate that enzymes also involved in phenylpropanoid biosynthesis

**Table S5. The differentially expressed of disease resistance related genes in canker pathogens inoculated poplars.**

|  | BdoA_Xy | BdoA_Ph | VsoC_Xy | VsoC_Ph |
| --- | --- | --- | --- | --- |
| Disease resistance genes | **Potri.019G098500 -2.62**  **Potri.001G444000 -3.45**  Potri.001G427600 -2.24  Potri.005G042900 1.07  Potri.017G143400 1.55  **Potri.011G040800 -2.34**  **Potri.014G010700 -1.68**  Potri.012G083700 -3.08  Potri.018G135600 1.11  Potri.014G011600 1.22  Potri.017G133700 1.08  Potri.001G425900 -2.59  **Potri.T045300 1.11**  Potri.005G061900 1.34 | **Potri.019G098500 -3.30**  Potri.005G042200 -2.23  **Potri.018G080700 1.95**  **Potri.019G097800 1.68**  **Potri.T077200 ---**  Potri.019G097100 1.44  **Potri.014G010700 -2.12**  **Potri.019G113700 3.72**  **Potri.019G098700 2.02**  **Potri.019G098900 2.24**  **Potri.019G097500 2.79 Potri.017G136900 1.37** | Potri.019G097500 2.86  Potri.017G133700 2.44  Potri.T096100 2.85  Potri.003G055600 2.39  Potri.T105500 2.80  **Potri.011G040800 -2.82**  Potri.001G025400 2.14  **Potri.019G113700 3.84**  Potri.018G080700 3.43  Potri.018G135600 1.94  Potri.T028700 1.81  **Potri.017G136900 2.43**  **Potri.T045300 2.08**  **Potri.T025800 2.36**  Potri.007G039100 1.48  Potri.003G200200 1.95 | **Potri.019G098500 -4.25**  **Potri.001G444000 -3.33**  **Potri.018G080700 2.77**  **Potri.019G097800 1.99**  **Potri.T077200 -3.91**  **Potri.011G040800 -1.15**  **Potri.014G010700 -1.23**  **Potri.019G113700 4.29**  **Potri.019G098700 2.49**  **Potri.019G098900 2.88 Potri.019G097500 3.42**  Potri.001G422800 1.44  **Potri.T045300 1.48**  **Potri.T025800 1.56**  Potri.001G427500 1.66  Potri.001G444500 -2.92  Potri.T065900 1.33  Potri.003G134600 -2.52  Potri.001G427700 1.72  Potri.019G002800 1.69  Potri.T025300 1.83  Potri.012G032400 -1.36  Potri.014G007900 1.61  Potri.019G036900 -2.99  Potri.001G025400 1.25  Potri.014G011600 1.53  Potri.014G009300 1.32  Potri.014G009400 1.00  Potri.017G133700 1.70  Potri.017G136900 1.90  Potri.T116200 2.82  Potri.003G055600 1.85  Potri.003G200200 1.64 |
| Peroxidase | **Potri.005G195700 -3.54**  **Potri.006G129900 1.44**  Potri.008G110600 -5.59  Potri.005G135300 -2.99  **Potri.001G011000 -5.62**  **Potri.001G011300 -4.63**  **Potri.016G125000 -5.30**  **Potri.004G144600 1.66**  **Potri.010G134500 -5.10**  **Potri.007G053400 -3.29**  **Potri.001G013000 -4.34**  **Potri.014G143200 -3.45**  **Potri.017G037900 -3.03**  **Potri.003G214900 -5.52**  **Potri.013G156500 -3.30**  **Potri.004G134800 1.66**  **Potri.001G011200 -4.83**  Potri.005G072800 1.17  Potri.001G351000 -1.36  Potri.002G018000 -3.19 |  | **Potri.005G195700 -4.30**  **Potri.006G129900 2.38**  **Potri.013G083600 -5.85**  **Potri.016G058200 -4.30**  **Potri.001G011000 -4.16**  **Potri.001G011300 -4.62**  **Potri.016G125000 -3.79**  **Potri.004G144600 3.03**  **Potri.010G134500 -5.35**  **Potri.007G053400 -3.72**  **Potri.001G013000 -2.88**  **Potri.014G143200 -4.49**  **Potri.017G037900 2.05**  **Potri.003G214900 -4.94**  **Potri.013G156500 -3.67**  **Potri.004G134800 3.08**  **Potri.001G011200 -3.41**  Potri.006G069600 2.43  Potri.017G038100 1.62  Potri.017G064100 -7.05 | **Potri.005G195700 -1.51**  **Potri.006G129900 1.37**  **Potri.015G003500 -1.48**  **Potri.016G058200 -1.71**  **Potri.001G011000 -1.92**  **Potri.001G011300 -2.23**  **Potri.016G125000 -4.87**  Potri.005G195600 -1.03  Potri.001G011500 ---  Potri.015G003600 -1.59 |
| Polyphenol oxidase | **Potri.001G388900 -4.79**  **Potri.011G047300 -6.34**  **Potri.011G047200 -2.10**  **Potri.001G387900 -2.37** | Potri.001G388100 -1.93  **Potri.011G047300 -2.69**  **Potri.001G387900 -1.27** | **Potri.001G388900 -5.26**  **Potri.011G047300 -7.05** | **Potri.001G388900 -2.46**  **Potri.011G047300 -7.88**  **Potri.011G047200 -2.10**  Potri.001G388400 -1.58  Potri.001G388200 -3.10  Potri.001G388100 -2.63  Potri.T062200 -2.30  Potri.001G388600 -2.57  Potri.T061900 -3.21 |

Notation: Bold indicates that DE genes co-expressed in more than one treatment.

**Table S6. Differentially expressed WRKY family genes in phloem and xylem in poplar infected by *Botryosphaeria* and *Valsa* pathogen.**

|  | Bdo_Xy vs Ctrl_Xy | | Bdo_Ph vs Ctrl_Ph | | Vso_Xy vs Ctrl_Xy | | Vso_Ph_Ctrl_Ph | |
| --- | --- | --- | --- | --- | --- | --- | --- | --- |
|  | Gene and expression | Subfamily | Gene and expression | Subfamily | Gene and expression | Subfamily | Gene and expression | Subfamily |
| WRKY transcription  factor | **Potri.014G155100 -3.81**  **Potri.002G164400 2.13**  Potri.002G228400 -2.25  Potri.004G007500 -2.64 | WRKY31  WRKY22  WRKY6  WRKY6 |  |  | **Potri.014G155100 -4.42**  **Potri.002G164400 4.18**  Potri.016G083600 -2.67 | WRKY31  WRKY22  WRKY44 | **Potri.014G155100 -2.38**  Potri.003G132700 -4.52  Potri.014G090300 -3.34 | WRKY31  WRKY22  WRKY22 |
| Probably WRKY transcription factor | **Potri.016G128300 -2.36**  Potri.018G008500 -2.46  Potri.018G019700 -4.06  **Potri.015G099200 -3.87**  Potri.016G083600 -1.70  Potri.001G208600 -2.39  Potri.010G147700 -1.55  Potri.017G079500 -2.20  **Potri.003G111900 2.57**  **Potri.002G195300 1.72**  **Potri.014G090700 -4.70**  **Potri.001G044500 -1.77**  **Potri.005G141400 -1.65**  **Potri.012G101000 -2.32**  **Potri.015G064100 -4.24**  **Potri.003G169100 -2.62**  **Potri.002G193000 -2.67**  Potri.007G047400 -1.46  Potri.001G058800 -3.32 | WRKY33  WRKY6  WRKY40  WRKY75  WRKY26  WRKY9  WRKY48  WRKY72  WRKY47  WRKY69  WRKY56  WRKY40  WRKY7  WRKY75  WRKY72  WRKY75  WRKY23  WRKY15  WRKY75 | **Potri.016G128300 3.02**  **Potri.006G105300 2.28**  **Potri.014G096200 2.44**  **Potri.015G099200 -1.49**  Potri.014G119800 -1.12  **Potri.003G138600 2.91**  Potri.001G092900 3.10 | WRKY33  WRKY33  WRKY53  WRKY75  WRKY85  WRKY41  WRKY41 | Potri.018G008500 -3.27  Potri.011G169300 1.79  **Potri.014G096200 3.99**  **Potri.015G099200 -5.23**  **Potri.013G153400 2.30**  **Potri.003G138600 2.26**  **Potri.001G352400 -5.50**  **Potri.011G007800 -5.11**  **Potri.014G111900 -2.42**  **Potri.002G195300 2.01**  **Potri.014G090700 -4.52**  **Potri.001G044500 -1.92**  **Potri.005G141400 -2.33**  **Potri.012G101000 -3.47**  **Potri.015G064100 -4.44**  **Potri.003G169100 -3.76**  **Potri.002G193000 -3.44**  Potri.003G182200 2.51  Potri.014G118200 -1.81  Potri.002G059100 -4.19  Potri.005G203200 -6.42  Potri.011G079300 -5.28  Potri.004G007500 -3.18  Potri.T043800 -1.89  Potri.001G328000 -1.69 | WRKY11  WRKY2  WRKY53  WRKY75  WRKY33  WRKY41  WRKY28  WRKY31  WRKY47  WRKY69  WRKY56  WRKY40  WRKY7  WRKY75  WRKY72  WRKY75  WRKY23  WRKY40  WRKY23  WRKY28  WRKY28  WRKY28  WRKY31  WRKY75  WRKY75 | **Potri.016G128300 3.44**  **Potri.006G105300 2.57**  **Potri.014G096200 4.01**  Potri.005G085200 1.98  **Potri.013G153400 2.57**  Potri.013G090300 1.94  **Potri.001G352400 -1.81**  **Potri.011G007800 -2.01** | WRKY33  WRKY33  WRKY53  WRKY51  WRKY33  WRKY70  WRKY28  WRKY31 |
| Regulation | 20 downregulated，3 upregulated | | 2 downregulated，5 upregulated | | 21 downregulated，7 upregulated | | 5 downregulated，6 upregulated | |

Notation：The co-expressed WRKY genes in more than one comparison were in bold and were listed in same row. The WRKY genes were differential regulated in two or more treatments were underlined.

**Table S7. The NSCs content in poplar stem and roots.**

| Tissues | NSCs Content (mg g^-1^) | 3 DAI | | 7 DAI | | 11 DAI | |
| --- | --- | --- | --- | --- | --- | --- | --- |
|  |  | Control | Inoculation | Control | Inoculation | Control | Inoculation |
| Stem phloem | Soluble sugars | 37.6±4.0 | 71.3±12.3 ** | 22.4±6.7 | 39.1±9.6 * | 50.0±9.5 | 70.7±17.4 * |
|  | Starch | 49.5±10.5 | 63.8±17.0 | 40.9±11.7 | 43.8±9.5 | 67.1±13.9 | 62.3±9.7 |
| Stem xylem | Soluble sugars | 28.9±10.8 | 26.5±1.3 | 18.9±4.1 | 17.9±2.3 | 24.1±3.5 | 18.6±3.2 * |
|  | Starch | 25.3±3.0 | 30.0±4.2 | 31.7±7.7 | 31.0±5.5 | 36.7±7.8 | 42.6±3.8 |
| Root | Soluble sugars | 24.4±9.2 | 20.0 ±1.6 | 27.6±3.2 | 29.4±6.8 | 53.0±15.2 | 33.6±4.8 * |
|  | Starch | 49.9±9.0 | 32.3±5.5 * | 20.7±6.8 | 29.2±6.8 | 33.9±1.7 | 45.4±8.4 |

Notation: The values are “Mean (SE)” in this table. Asterisks indicate treatments that significantly differed between the inoculation and control at the same timepoint (ANOVA; *: *P* < 0.05; **: *P* < 0.01; n = 6).
